# Azithromycin promotes proliferation, and inhibits inflammation in nasal epithelial cells in primary ciliary dyskinesia

**DOI:** 10.1038/s41598-023-41577-5

**Published:** 2023-09-02

**Authors:** Zofia Varenyiova, Laura S. Rojas-Hernandez, Jacquelyn Spano, Vaclav Capek, Yael Rosenberg-Hasson, Tyson Holmes, Carlos Milla

**Affiliations:** 1 Department of Pediatrics, 2nd Faculty of Medicine, Charles University and Motol University Hospital, Prague, Czech Republic; 2 Center for Excellence in Pulmonary Biology, Stanford University, Palo Alto, CA USA; 3 Human Immune Monitoring Center, Stanford University, Stanford, CA USA

**Keywords:** Diseases, Medical research

## Abstract

Primary ciliary dyskinesia (PCD) is a genetic disorder associated with recurrent and chronic respiratory infections due to functional defects of motile cilia. In this study, we aimed to elucidate inflammatory and proliferative responses in PCD respiratory epithelium and evaluate the effect of Azithromycin (AZT) on these responses. Airway basal cells (BCs) were isolated from nasal samples of Wild-type (WT) epitope of healthy donors and PCD donors with bi-allelic mutations in DNAH5, DNAH11 and CCDC39. Cells were expanded in vitro and stimulated with either Lipopolysaccharide (LPS) or vehicle control. Post stimulation, cells were treated with either Azithromycin (AZT) or vehicle control. Cell proliferation was imaged in real-time. Separately, BCs from the same donors were expanded and grown at an air–liquid interface (ALI) to generate a multi-ciliated epithelium (MCE). Once fully mature, cells were stimulated with LPS, AZT, LPS + AZT or vehicle control. Inflammatory profiling was performed on collected media by cytokine Luminex assay. At baseline, there was a significantly higher mean production of pro-inflammatory cytokines by CCDC39 BCs and MCEs when compared to WT, DNAH11 and DNAH5 cells. AZT inhibited production of cytokines induced by LPS in PCD cells. Differences in cell proliferation were noted in PCD and this was also corrected with AZT treatment.

## Introduction

Human airway epithelium consists of several cell types with a population of multi-ciliated cells (MCEs) playing an important role in muco-ciliary clearance (MCC). MCC acts as a first line of defence against inhaled pathogens^1^. Primary ciliary dyskinesia (PCD) is a rare genetic disease characterized by a dysfunction, or, more rarely, reduced number of motile cilia in respiratory epithelial cells^2^. This results in impaired MCC, which establishes the conditions for bacterial overgrowth, and hence chronic and recurrent respiratory infections. Clinically, patients with PCD suffer from chronic rhinosinusitis, recurrent otitis media leading to conductive hearing loss, chronic wet cough, chronic bronchitis, bronchopneumonia and progressive bronchiectasis, and laterality defects^3^.

PCD is genetically heterogenous—mutations in up to 63 genes have been associated with motile ciliopathies^2, 4–6^. Genetic variability of the condition determines its diverse clinical picture varying between relatively mild phenotype to patients with chronic respiratory failure requiring lung transplants^7^. Genes involved in PCD can be categorized into distinct subgroups from the structural ciliary components affected and the resulting functional defects. Further, it is becoming clearly apparent that there is a clinical correlate between specific categories and disease severity. Genes that result in absent inner dynein arms with microtubular disorganization (IDA/MTD) (e.g. CCDC39, CCDC40) are emerging as associated with more severe disease. Patients with mutations in CCDC39 are significantly younger at diagnosis, have significantly reduced FEV_1_ and worse radiographic disease when compared to mutations in DNAH11 and DNAH5^8–10^. In contrast, mutations in DNAH11 are associated with lower frequency of neonatal respiratory distress syndrome and better outcome, while variants in DNAH5 lead to variable clinical symptoms^10^.

Currently, there is no disease-specific therapy available for PCD, much less effective disease modifying therapeutics. Therapeutic recommendations are primarily focused on symptom control and delaying complications and disease progression. Management of the condition mainly consists of regimens that include episodic antibiotics, and airway clearance facilitated by inhaled bronchodilators, mucolytics and respiratory physiotherapy^11^. Azithromycin (AZT) maintenance therapy has been proposed to control inflammation and reduce respiratory exacerbations in PCD^12^. Although increased inflammatory markers have been reported in the sputum of PCD patients^13, 14^, and this associates with lung disease^15^, the inflammatory environment in the PCD respiratory tract has not been fully characterized. It has been suggested that CXCL8/IL8-drives neutrophilic accumulation in the airway of PCD patients and might then play a key role in inducing chronic airway inflammation^13, 14, 16, 17^, but the source of this chemotactic stimuli has not been fully characterized.

Basal airway epithelial cells (BC) are a multipotent stem cell population with key roles in airway epithelium homeostasis and repair^18^. Despite their importance, little is known about BCs contribution to pathogenic airway remodelling in the context of PCD and to what extent changes in cellular phenotype are driven by their defective PCD gene vs. the inflammatory milieu due to the chronic infections that patients experience.

Our aim was to further investigate the inflammatory and healing responses in PCD epithelia. We hypothesize that respiratory cells from PCD patients demonstrate heightened inflammatory responses and disrupted proliferation. Further, we hypothesize that Azithromycin modifies immune mechanisms contributing to chronic inflammation in PCD and promotes epithelial barrier renewal. To our knowledge, this study is the first to carry this evaluation in primary cells from PCD patients with different affected genes and in comparison to wild type cells from healthy donors. We furthered our observations by evaluating the effect of azithromycin on the respiratory epithelium in PCD.

## Materials and methods

### Cell expansion and culture

Human nasal epithelial cells (HNECs) from WT and PCD patients with mutations in *DNAH5, DNAH11* and *CCDC39* (Table 1) were obtained by nasal brushings and processed as we have previously described^19–21^ Nasal cell collections were performed under a protocol approved by the Stanford University Institutional Review Board (Protocol #42710). Informed consent was obtained from all subjects or their legal guardians. All methods were performed in accordance with the Stanford University guidelines and regulations. Cells were then seeded into either 96-well plate for BC proliferation experiments, or collagen-coated Transwell inserts (0.33 cm^2^, 0.4 μm pore size, Corning 3470, Thermo Fisher Scientific Inc.) for expansion and air–liquid interface (ALI) culture for experiments assessing multiciliated epithelium (MCE) responses.

**Table 1:** Clinical characteristics by genotype the of PCD patients that provided cell samples for the study.

	DNAH5	CCDC39	DNAH11	WT1	WT2
Mutation	c.10815del c.13309del	c.357 + 1G > C c.357 + 1G > C	c.7904_7914 + 643del c.7904_7914 + 643del	N/A	N/A
Age at sample	3 years	18 years	6 months	40 years	52 years
Age at diagnosis	2 years	13 years	6 months	N/A	N/A
Infection present at time of collection	No	No	No	No	No
Treatment with azithromycin	No	No	No	No	No
Treatment with corticosteroids	Inhaled yes	No	No	No	No
FEV1 at diagnosis	NA	80%-pred	NA	100%-pred	102%-pred

### Stimulation experiments and growth analysis

First, BCs from the WT and PCD donors were seeded in collagen coated Transwell inserts (Corning 3470, Thermo Fisher Scientific Inc.). Cells were expanded with Pneumacult EX Plus (05040, Stemcell Technologies) for 7 days and upon confluency, ALI conditions were established by replacing media only in the basal chamber with Pneumacult ALI (05001, Stemcell Technologies) and removing the apical media. Media was changed every other day for 21 days to allow cells to fully differentiate into an MCE. Differentiation was verified by evaluating the samples for the presence of multiciliated cells under phase contrast microscopy and evaluation of ciliary motility by high-speed video microscopy, as we have previously described^20^ (representative images for each donor presented in Supplementary material), cells were treated on their basolateral surface with vehicle control, lipopolysaccharide (LPS) (20 µg/mL), AZT (10 µg/mL) or LPS + AZT for 48 h. Basal chamber media from all experimental conditions were collected at baseline (at 21 days upon differentiation) and after 48-h of treatment and stored at − 80 °C for later batch analysis.

In a second set of experiments, BCs from the same WT and PCD patients were seeded in collagen-coated 96-well plates (1000 cells/well). The BCs were allowed to attach overnight with 100 µL/well of Pneumacult EX Plus (05040, Stemcell Technologies). Plates were then transferred to an Incucyte^®^ SX1 Live-Cell Analysis System (Sartorius, Goettingen, GmbH) for real-time monitoring of cell growth by phase-contrast imaging at 100× (5 scans of each well/hour). Images were automatically processed by the instruments software to generate label-free cell confluence estimates for each well (Incucyte^®^ Live-Cell Analysis System, Sartorius, Goettingen, GmbH). Random wells at different time points were selected to train the system and verify appropriateness of mask parameters.

Following initial proliferation, BCs were stimulated with 20 µg/mL of *E. coli* LPS (strain O111:B4, Catalog No. L4391, Millipore Sigma). After 24-h stimulation with LPS, cells were treated with Azithromycin (Azithromycin, CP-62993, Catalog No. S1835, Selleck Chemicals) (1 µg/mL or 10 µg/mL) for 48 h. Supernatants were collected at baseline (after 24-h incubation with vehicle control), post 24-h LPS stimulation, and post 48-h AZT treatment, and stored at – 80 °C until ready for batch analysis.

### Cytokine analysis

Supernatants from BCs and basal media from MCEs were assayed for inflammatory profiling at baseline and 48-h after treatments by 42-Luminex human inflammatory array (Luminex-EMD Millipore Human 80 Plex kit, Millipore Corporation, Burlington, MA). The full list of cytokines is shown in Supplementary Table S1. This assay was performed by the Human Immune Monitoring Core Center at Stanford University and run according to a protocol optimized in house^22^. Each sample was measured with duplicate replicates for BCs and single replicates for MCEs. Custom Assay Chex control beads were purchased and added to all wells (Radix BioSolutions, Georgetown, Texas). Wells with a bead count < 50 were flagged, and data with a bead count < 20 were excluded.

### Statistical analysis

Cytokine production was analyzed by generalized maximum entropy estimation regression (GMEE)^23^ and quantile regression^24^ with p-values adjusted^25, 26^ to control the false discovery rate at 5% across all cytokines within a comparison. Regression analyses were adjusted for covariates of nonspecific binding and, where applicable for each plate and PCD gene. GMEE assumes approximate variance homogeneity among groups; where this assumption is violated, results must be interpreted with caution. All GMEE analyzes were performed in SAS^®^ (SAS^®^ Institute, Cary, North Carolina, USA). Data in graphs are adjusted for non-specific binding. Cell proliferation was analysed by a linear mixed-effects model with interactions^27^. As a random effect a two-level nested factor of subject and experiment was used. Interactions were assumed among the time variable, the group factor (patient/healthy control) and the treatment itself. The modelled variable was used as log-transformed. p-values were computed via the Satterthwaite's degrees of freedom method.

## Results

### Multi-ciliated cells

#### Respiratory epithelium in PCD has pro-inflammatory characteristics

**Figure 1:**
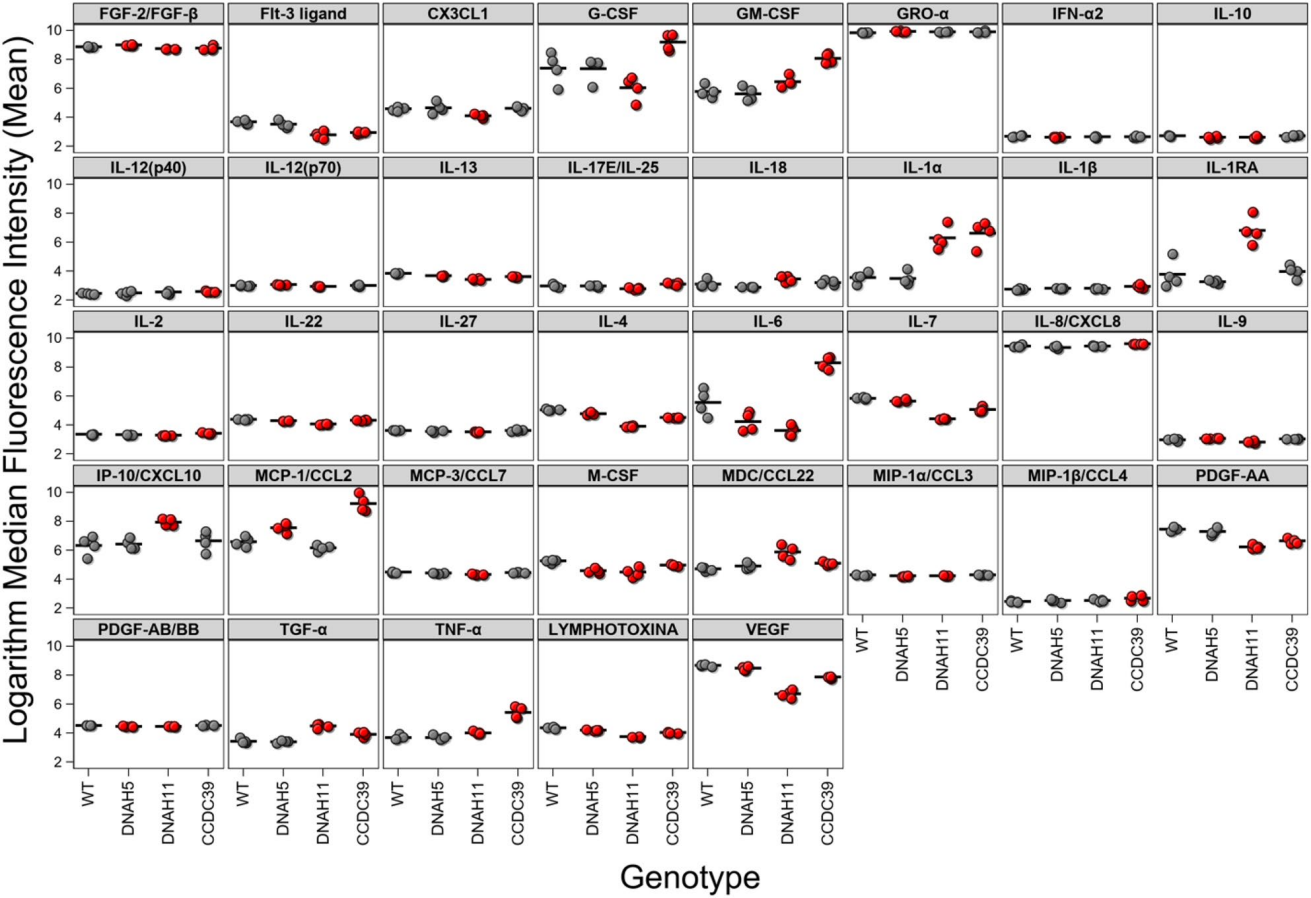
Cytokine production, measured as median fluorescence intensity (MFI), by MCEs under unstimulated baseline conditions for each genotype. Mean (bar) MFI for the samples from each genotype studied.

First, we aimed to characterize the inflammatory environment in respiratory epithelium of PCD patients. Our experiments showed that under unstimulated conditions, MCEs derived from PCD patients had higher mean production of certain pro-inflammatory cytokines (IL-1α, IL-6, IL-8/CXCL8, TNF-α) and growth factors for granulocytes (GM-CSF) and T cells (IL-2) than WT (p < 0.05) (Fig. 1). Furthermore, MCEs with mutations in CCDC39 showed significantly increased mean production of pro-inflammatory cytokines (IL-1α, IL-6, IL8/CXCL8, TNF-α, MCP-1/CCL2) when compared to DNAH11 and DNAH5 (p < 0.05). These findings indicate that PCD airway cells have heightened inflammatory activity, with CCDC39 demonstrating the strongest responses, which corresponds to the known disease severity observed in patients with mutations in this gene. To model the response to acute infection, PCD MCEs were stimulated with LPS. As expected, LPS promoted significant increase in the mean production of pro-inflammatory cytokines in ciliated cells from PCD patients when compared to vehicle control (p < 0.05) (Supplementary Fig. S1).

#### Low-dose AZT inhibits inflammation in PCD

**Figure 2:**
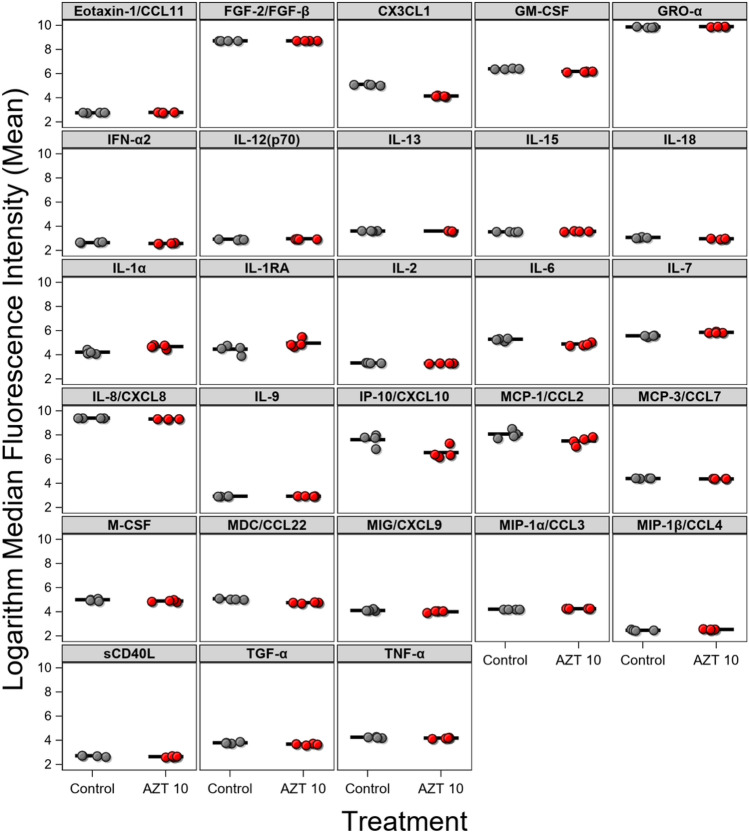
Cytokine production in PCD MCEs under unstimulated conditions treated with vehicle control (gray) vs AZT 10 µg/mL (red). Mean (bar) MFI for the samples, only cytokines with statistically significant differences between control and treatment presented.

**Figure 3:**
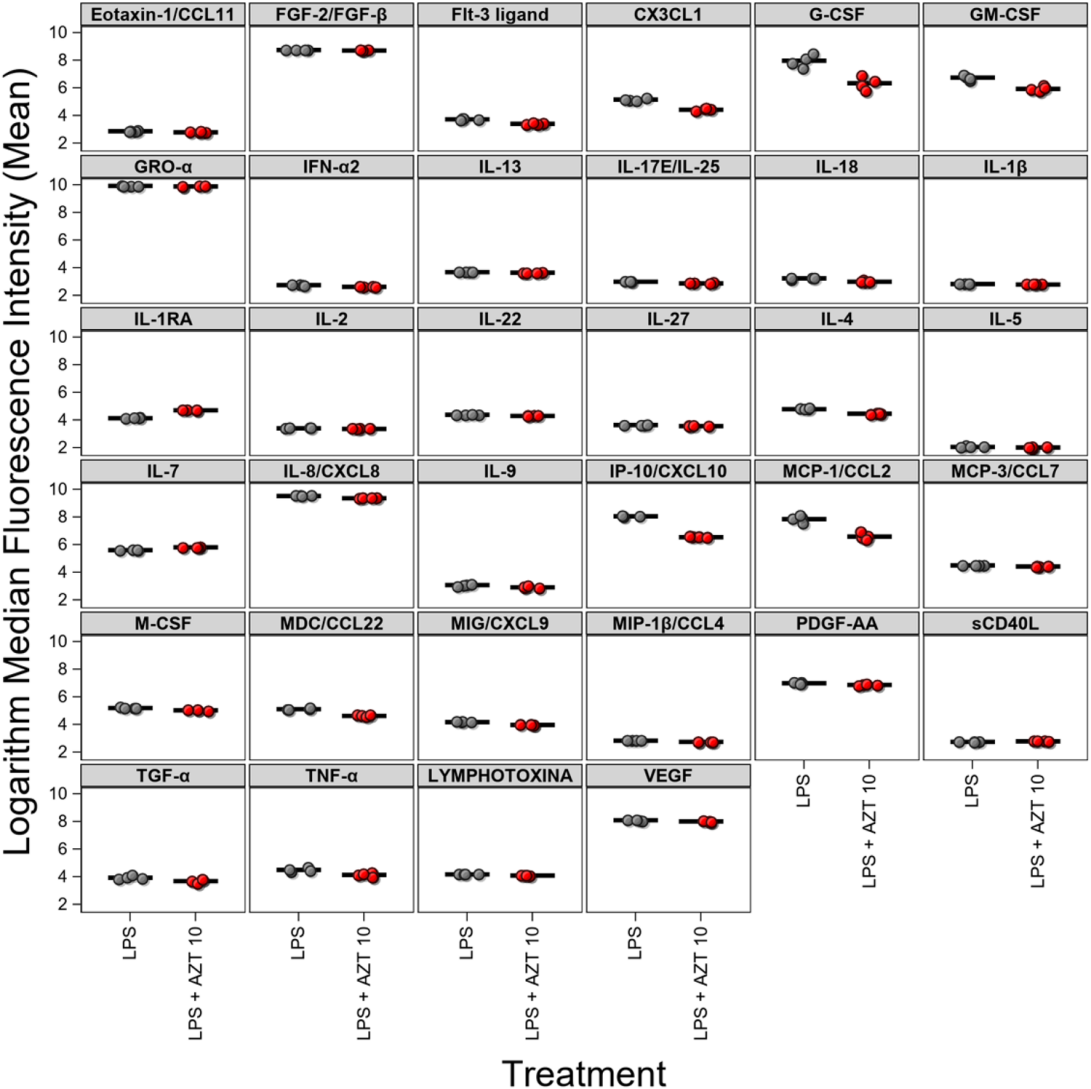
Cytokine production in PCD MCEs treated with LPS (gray) vs LPS + AZT 10  µg/mL (red). Mean (bar) MFI for the samples, only cytokines with statistically significant differences between control and treatment presented.

To evaluate the effect of AZT on inflammatory responses in PCD, MCEs were treated with AZT, LPS or both. Treatment with AZT (10 µg/mL) significantly inhibited mean production of pro-inflammatory cytokines and hematopoietic growth factors (IL-6, IL-8/CXCL8, MCP-1/CCL2, TNF-α, TGF-α, IL-2, GM-CSF) in PCD MCEs both at baseline (Fig. 2) and after co-treatment with LPS (p < 0.05) (Fig. 3). Furthermore, AZT (10 µg/mL) promoted increase in the mean production of anti-inflammatory cytokine IL-1RA in these cells. These data suggest that AZT promotes homeostasis in the respiratory mucosa in PCD by both inhibition of the inflammatory cytokine production and stimulation of anti-inflammatory responses.

### Basal cells

**Figure 4:**
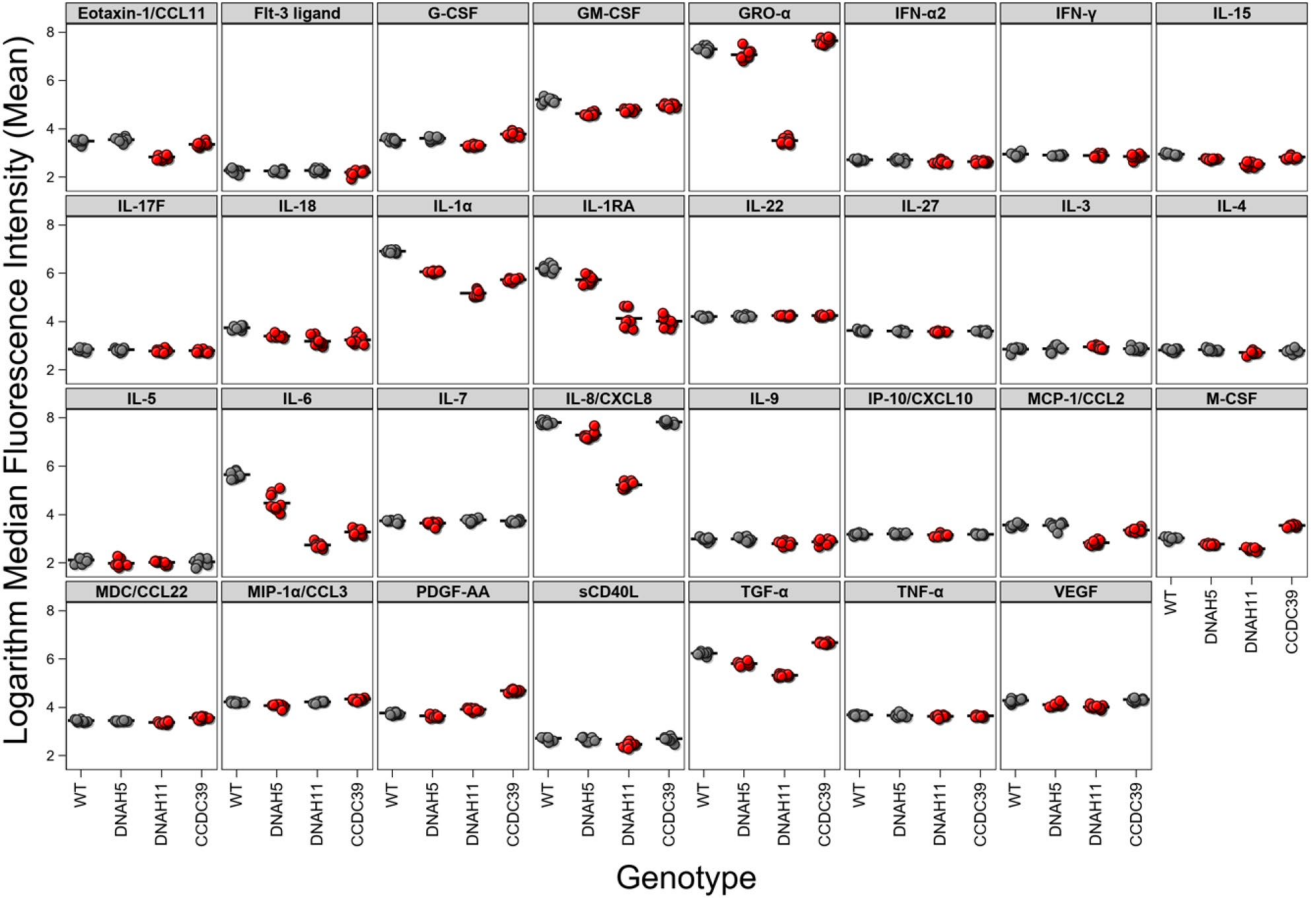
Cytokine production by BCs under unstimulated baseline conditions for each genotype. Mean (bar) MFI for the samples from each genotype studied.

Next, we aimed to evaluate inflammatory responses in BCs and assess the effects of AZT on inflammation and epithelial cell regeneration in PCD. Similar to MCEs, PCD BCs showed increased mean production of pro-inflammatory cytokines and growth factors for hematopoietic cells (IFN-α2, TGF-α, GRO-α, G-CSF, M-CSF) at baseline when compared to WT (p < 0.05) (Fig. 4), with the strongest inflammatory response in CCDC39 BCs (p < 0.05) (Fig. 4). Treatment with LPS resulted in further increase in the production of pro-inflammatory cytokines in PCD BCs when compared to vehicle control (Supplementary Fig. S2). Similar to our experiment with differentiated MCEs, treatment with 1 µg/mL AZT induced a reduction in production of pro-inflammatory cytokines in LPS-stimulated BCs from PCD patients (Supplementary Fig. S3).

#### AZT promotes proliferation of respiratory BCs in patients with PCD

**Figure 5:**
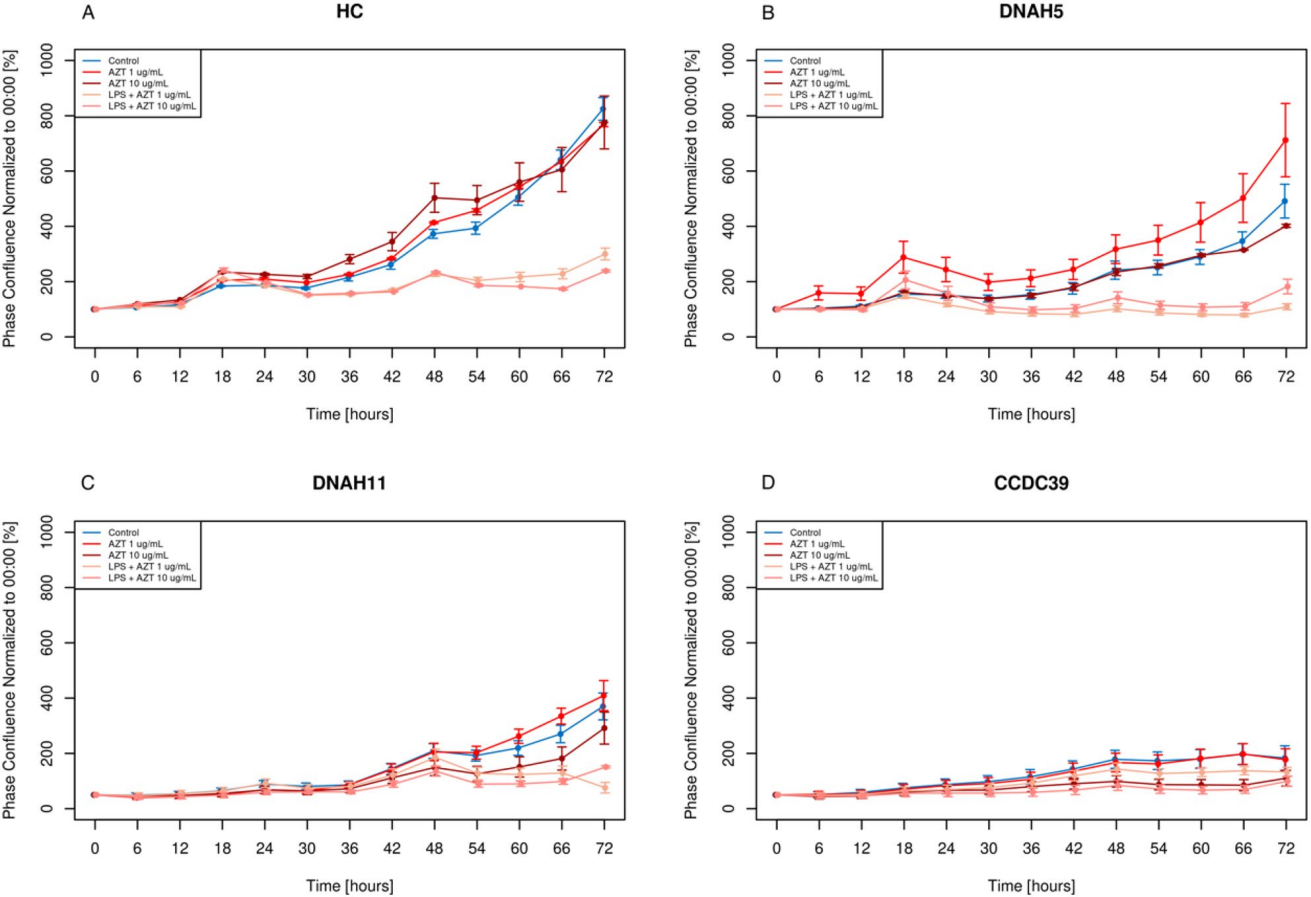
Proliferation of BCs in WT and PCD under baseline and treatment conditions. The progression of BCs towards confluence was monitored for 72 h in WT (HC, healthy control) (**A**), DNAH5 (**B**), DNAH11 (**C**) and CCDC39 (**D**) under baseline unstimulated control conditions (blue) and AZT (red) or AZT + LPS (pink) treatment.

To evaluate the effect of AZT on BCs proliferation, we conducted real-time monitoring by time-lapse image capture with Incucyte^®^ SX1 Live-Cell Analysis System (Sartorius). Treatment with 1 µg/mL AZT significantly promoted cell proliferation in both WT and PCD BCs when compared to treatment with vehicle control (p = 0.02) (Fig. 5). In contrast, higher dose of AZT (10 µg/mL) significantly inhibited cell growth of PCD BCs when compared to treatment with vehicle control (p < 0.00). As shown in Fig. 5, LPS has a significant detrimental effect inhibiting cell proliferation in BC of both PCD and WT (p < 0.01), irrespective of the treatment with AZT. These results indicate that low-dose AZT may promote epithelial barrier renewal in the respiratory mucosa of PCD patients and provide rationale for AZT maintenance therapy.

## Discussion

The airway epithelium plays an important role as an innate defence mechanism in the airways—it represents a structural barrier, provides removal of inhaled microbes and pollutants by muco-ciliary clearance, and produces anti-microbial peptides and cytokines^28, 29^. One of the key players in the maintenance of airway epithelial homeostasis are the BCs—a subpopulation of airway progenitor cells that are crucial for both physiological epithelial turnover and post-injury repair^30^.

In PCD, the accumulation of bacteria in the stagnating mucus leads to chronic inflammation which disrupts the integrity of epithelial barrier. PCD patients suffer from recurrent acute and chronic respiratory infection predominantly caused by bacteria such as *Hemophilus influenzae*, *Streptococcus pneumoniae*, *Moraxella catarrhalis*, *Staphylococcus aureus* and *Pseudomonas aeruginosa*^31^.

In this work, we aimed to investigate in vitro the inflammatory environment in airway epithelia in PCD. Our data demonstrates under unstimulated conditions pro-inflammatory cytokine production by both BC and MCE PCD cells when compared to WT cells. Previously published data on PCD inflammatory cytokine environment is limited. A few studies have pointed to an enhanced IL-8/CXCL8 production in peripheral blood monocytes and in sputum from patients with PCD^13, 14, 16^. We detected increased pro-inflammatory cytokine production in PCD epithelial cells, including chemoattractant factors for neutrophils such as GROA/CXCL1 and CXCL8/IL8^32, 33^; key pro-inflammatory cytokines—IL1A, IL6 and TNFA^34^; and molecules that function as growth factors for neutrophils, macrophages, dendritic cells, and fibroblasts (GMCSF, GCSF, MCSF, FLT3L, PDGFAA). Our experiments suggest an underlying pro-inflammatory response in the PCD respiratory epithelium in the absence of an acute bacterial infection.

In addition, we find PCD BCs to have defective proliferative responses as evidenced by a delayed progression towards confluence. Although it could be argued that this could be related in part to their underlying pro-inflammatory phenotype, as it was rescued by AZT for the PCD cells, the lack of a response to the detrimental effects of LPS argues for other possible factors being at play. In other contexts, AZT has been shown to enhance epithelial repair by scratch-wound assay^35^, and promote barrier integrity^34^. Further investigation of these effects and potential mechanisms behind the dysfunctional proliferation noted in PCD cells are ongoing.

Recent study by Shoemark and Rubo et al. (2021), who applied topological data analysis approach on a group of almost 400 patients with PCD-related mutations demonstrated that disease symptoms vary from severe in CCDC39, variable in DNAH5 to mild in DNAH11^10^. From our findings, we propose that this clinical disease severity can be in part explained by the severity of inflammation being also genotype-associated, as cells with a mutation in CCDC39 exhibited highest mean production of pro-inflammatory cytokines when compared to DNAH5, DNAH11 and WT. However, CCDC39 sample in this study was collected from an adolescent patient, so it is important to consider a possible role of age-dependent factors in the severity of inflammation, such as epigenetics. Still, our findings are in agreement with accumulating evidence for a genotype-determined diversity of disease severity in PCD.

Moreover, stimulation with LPS to mimic the conditions induced by bacterial infection led to further increase in the levels of pro-inflammatory cytokines in PCD cells, demonstrating a heightened response. To the best of our knowledge, no similar experiment assessing cytokine production and proliferation capacity in PCD epithelial cell cultures has been performed to this date. There are a few studies that assessed NO production in HNECs derived from PCD patients in a response to bacterial infection and treatment with pro-inflammatory cytokines, however, with inconclusive results^37, 38^.

In our work, we showed that AZT might play a crucial role in the maintenance of epithelial barrier integrity and inhibition of chronic inflammation in the respiratory mucosa of PCD patients. AZT is known for its antimicrobial and immunomodulatory properties, ability to inhibit bacterial quorum sensing and biofilm formation^36, 39^. However, exact mechanisms by which AZT helps decrease the frequency of exacerbations in PCD are not fully understood^12^.

To assess the effects of AZT on the PCD airway, we treated cell cultures with 2 different concentrations of AZT. Our data showed that AZT at both doses tested can inhibit production of pro-inflammatory cytokines in PCD epithelium both at baseline and post treatment with LPS, and this irrespective of genotype. This effect was observed in both BCs and ciliated cells. Similarly, AZT-induced reduction of inflammatory cytokines in airway cells from lung allograft recipients was observed by Ling et al.^40^.

In our experiment on ciliated cells, AZT further induced increase in the mean production of immunoregulatory cytokine IL1RA. Besides functioning as a natural antagonist to IL1, IL1RA is reported to be involved in alveolar epithelial repair and elimination of bacteria from lungs^41^. Our results indicate that AZT modulates the cytokine production by respiratory epithelial cells and might help control inflammation in PCD.

Furthermore, several studies suggested that AZT might promote maintenance of respiratory epithelial barrier integrity. AZT has been reported to alter processing of tight junctions in respiratory epithelium and hence induce epithelial barrier maintenance in HNECs^42^. Slater et al. reported that AZT promoted re-epithelization in healthy human airway epithelium, while LPS lead to the loss of epithelial barrier integrity^36^. Correspondingly, our results showed significantly increased proliferation in PCD BCs after treatment with 1 µg/mL AZT when compared to vehicle control. In contrast, co-culture of BCs with LPS resulted in the inhibition of cell proliferation and cell growth that could not be rescued by AZT treatment. We speculate that the protective effect of AZT on the airway epithelium is limited to modulating the inflammatory responses and might be insufficient to enhance regenerative responses following a state of acute infectious exacerbation as modelled by our LPS experiments. Our ongoing work aims to explore the mechanisms at play in the epithelial barrier maintenance under the chronic inflammatory milieu present in PCD.

A limitation of our study is the small number of samples included. Collecting samples from multiple patients for each gene (DNAH5, DNAH11 and CCDC39) and at different ages would allow to verify our findings and better assess the variability in the responses seen, as well as permit separating donor characteristics (e.g. age, gender) from mutation specific effects. However, as PCD is a very rare disease, collecting a larger cohort of patients is challenging. Secondly, acute infection in this study was modelled in vitro using LPS rather than induced by direct exposure of cell culture to bacteria. Introducing bacterial infection might modify the cytokine spectrum produced by epithelial cells as well as the ability of AZT to inhibit inflammation. This forms the basis of additional ongoing studies. Lastly, in vitro models differ from in vivo conditions. However, RNA sequencing previously showed that ALI models are highly representative of the conditions in vivo, as the transcriptomic profiles were similar in 96% of the genes, when comparing cells from ALI culture and cells obtained directly from nasal brushings^43^.

## Conclusions

We identified increased inflammatory activity in PCD epithelial cells under unstimulated conditions in vitro when compared to WT cells*.* Our experiments render a mechanistic basis to clinical genotype–phenotype studies demonstrating differences in PCD, as the level of inflammation in CCDC39 cells proved to be significantly greater when compared to DNAH5, DNAH11 and WT. AZT treatment mitigated inflammation in both BCs and MCEs and promoted cell proliferation in PCD BCs, suggesting a beneficial effect on immune responses and epithelial barrier integrity in the PCD airway.

### Supplementary Information

Supplementary Video 1.

Supplementary Video 2.

Supplementary Video 3.

Supplementary Information.

Supplementary Video 4.

## Data Availability

The datasets generated and analyzed during the current study are available from the corresponding author on request.

## Abbreviations

AZT: Azithromycin
BCs: Basal cells
EOTAXIN/CCL11: Chemokine (C–C motif) ligand 11
FGF2/FGFB: Fibroblast growth factor 2/fibroblast growth factor beta
FLT3L: FMS-like tyrosine kinase 3 ligand
FRACTALKINE/CX3CL1: Chemokine (C-X3-C motif) ligand 1
GCSF: Granulocyte colony stimulating factor
GMCSF: Granulocyte–macrophage colony stimulating factor
GMEE: Generalized maximum entropy estimation
GROA/CXCL1: Chemokine growth-regulated protein alpha/chemokine (C-X-C motif) ligand 1
HNECs: Human nasal epithelial cells
IFNA2: Interferon alpha 2
IFNG: Interferon gamma
IL12P40: Interleukin 12p40
IL12P70: Interleukin 12p70
IL13: Interleukin 13
IL15: Interleukin 15
IL17A/CTLA8: Interleukin 17/cytotoxic T-lymphocyte antihen-8
IL17E/IL25: Interleukin 17E/interleukin 25
IL17F: Interleukin 17F
IL18: Interleukin 18
IL1A: Interleukin 1 alpha
IL1B: Interleukin 1 beta
IL1RA: Interleukin 1 receptor antagonist
IL2: Interleukin 2
IL22: Interleukin 22
IL3: Interleukin 3
IL4: Interleukin 4
IL4: Interleukin 4
IL6: Interleukin 6
IL7: Interleukin 7
IL8/CXCL8: Interleukin 8/chemokine (C-X-C motif) ligand 8
IL9: Interleukin 9
IP10/CXCL10: Interferon gamma-induced protein 10/chemokine (C-X-C motif) ligand 10
LPS: Lipopolisacharide
LYMPHOTOXINA: Lymphotoxin alpha
MCC: Mucociliary clearance
MCEs: Multi-ciliated epithelium
MCP1/CCL2: Monocyte chemoattractant protein 1/chemokine (C–C motif) ligand 2
MCP3/CCL7: Monocyte chemotactic protein 3/chemokine (C–C motif) ligand 7
MCSF: Macrophage colony stimulating factor
MDC/CCL22: Macrophage-derived chemokine/chemokine (C–C motif) 22
MFI: Median fluorescence intensity
MIG/CXCL9: Monokine induced by gamma/chemokine (C-X-C motif) ligand 9
MIP1A/CCL3: Macrophage inflammatory protein-1alpha/chemokine (C–C motif) ligand 3
MIP1B/CCL4: Macrophage inflammatory protein-1beta/chemokine (C–C motif) ligand 4
PCD: Primary ciliary dyskinesia
PDGFAA: Platelet-derived growth factor AA
SCD40L: Soluble CD40 ligand
TGFA: Transforming growth factor alpha
TNFA: Tumor necrosis factor alpha
WT: Wild type
