# Supplementary material for: Azithromycin promotes proliferation, and inhibits inflammation in nasal epithelial cells in primary ciliary dyskinesia
Source: Sci Rep. 2023 Sep 2;13:14453. doi: 10.1038/s41598-023-41577-5 (PMC10475097; doi:10.1038/s41598-023-41577-5)

**Supplementary material**

**Table S1**. List of cytokines analysed by 42-Luminex array. While certain molecules can be categorized according to their functions as predominantly pro-inflammatory cytokines and chemokines such as IL-1, IL-6, IL-8/CXCL8, TNF-α [32, 33, 35] or anti-inflammatory factors such as IL-1RA, IL10 [41]; many cytokines/chemokines have pleiotropic properties, functioning as both pro-inflammatory and immuno-regulatory molecules, and growth factors depending on tissue environment (GM-CSF, IL-3, IL-5) [1S].

|  |  |  |
| --- | --- | --- |
| sCD40L | IL-6 | MCP-1/CCL2 |
| EGF | IL-7 | MCP-3/CCL7 |
| Eotaxin/CCL11 | IL-8/CXCL8 | MDC/CCL22 |
| FGF-2/FGF-β | IL-9 | MIG/CXCL9 |
| Flt-3 ligand | IL-10 | MIP-1α/CCL3 |
| Fractalkine/CX3CL1 | IL-12(p40) | MIP-1β/CCL4 |
| GRO-α | IL-12(p70) | PDGF-AA |
| INF-α2 | IL-13 | PDGF-AB/BB |
| IFN-γ | IL-15 | RANTES/CCL5 |
| IL-1α | IL-17A/CTLA8 | TGF-α |
| IL-1β | IL-17E/IL-25 | TNF-α |
| IL-1RA | IL-17F | TNF-β/Lymphotoxin-a(LTA) |
| IL-2 | IL-18 | VEGF |
| IL-3 | IL-22 | G-CSF |
| IL-4 | IL-27 | GM-CSF |
| IL-5 | IP-10/CXCL10 | M-CSF |
|  |  |  |

**Table 1S references:**

1S. Dougan, M., Dranoff, G., Dougan, S.K., 2019. GM-CSF, IL-3, and IL-5 Family of Cytokines: Regulators of Inflammation. *Immunity* **50(4):**796-811. <https://doi.org/10.1016/j.immuni.2019.03.022>

**Figure S1. Cytokine production in PCD MCEs treated with vehicle control (grey) vs LPS (20 ug/ml) (red).** Mean (bar) MFI for the pooled PCD genotype samples, only cytokines with statistically significant differences between control and LPS treatment presented.


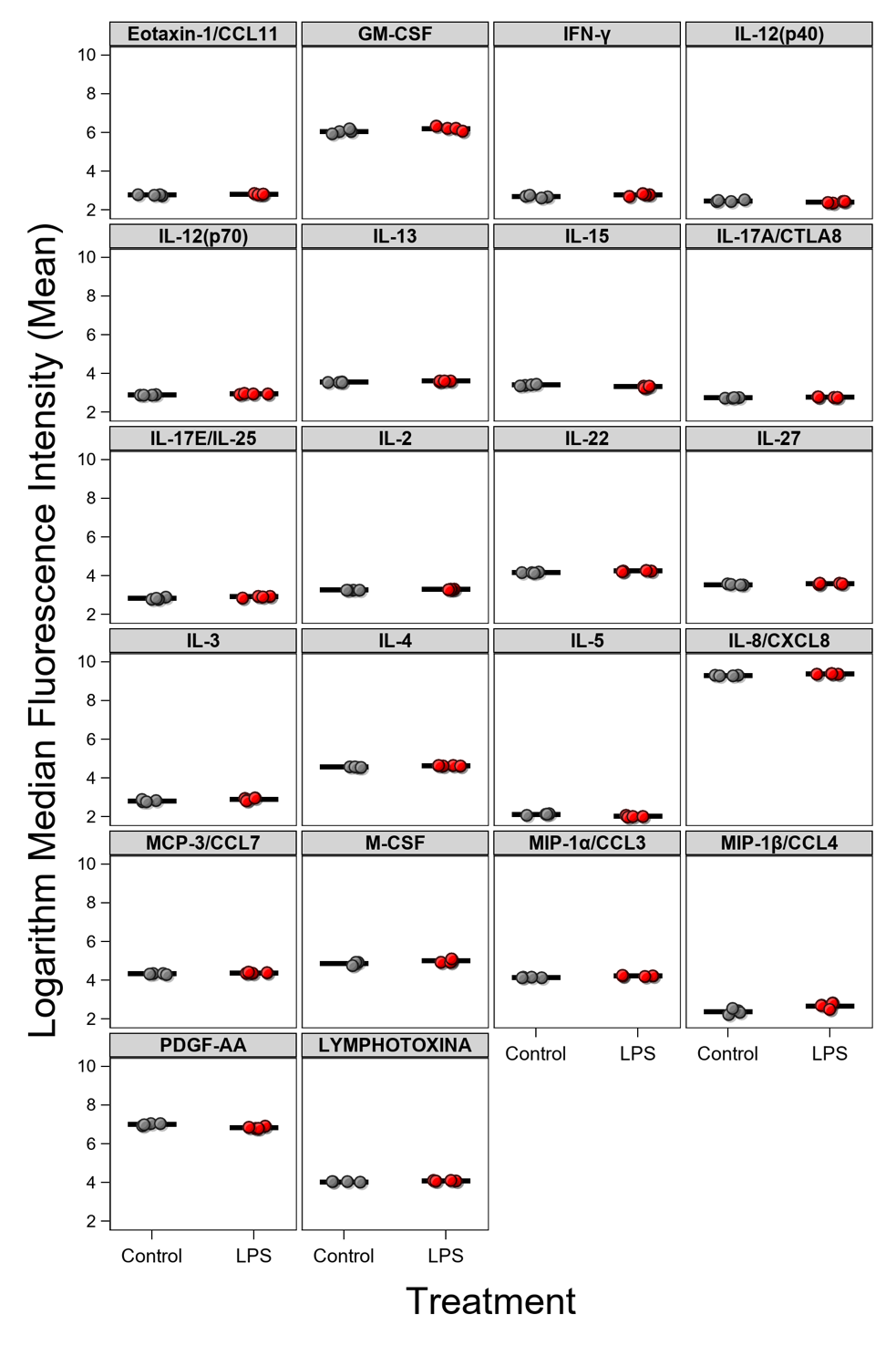


**Figure S2. Cytokine production in PCD BCs treated with vehicle control (grey) vs LPS (20 ug/ml) (red).** Mean (bar) MFI for the pooled PCD genotype samples, only cytokines with statistically significant differences between control and LPS treatment presented.


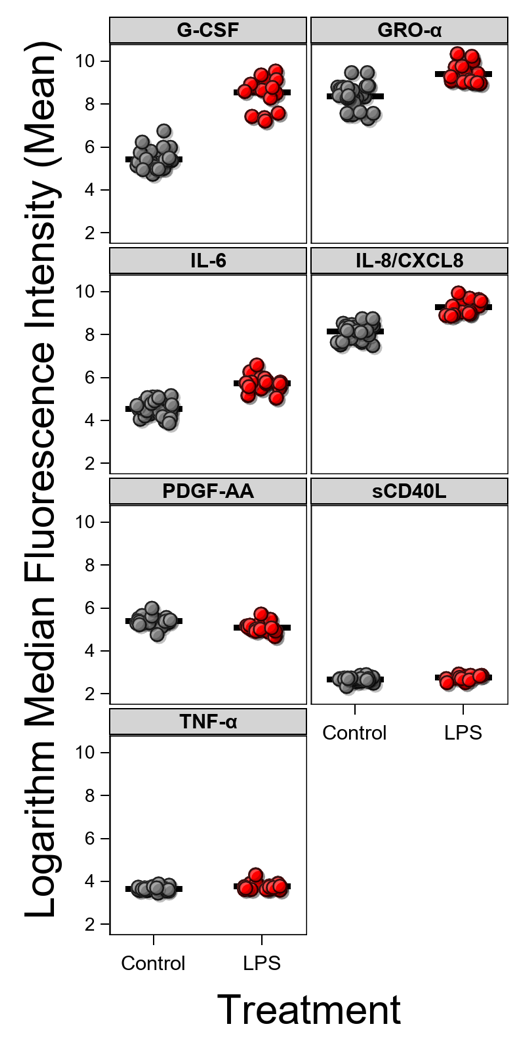


**Figure S3. Cytokine production in PCD basal cells treated AZT 1 ug/ml (grey) vs LPS + AZT** **1 ug/ml (red).** Mean (bar) MFI for the pooled PCD genotype samples, only cytokines with statistically significant differences between control and LPS treatment presented.


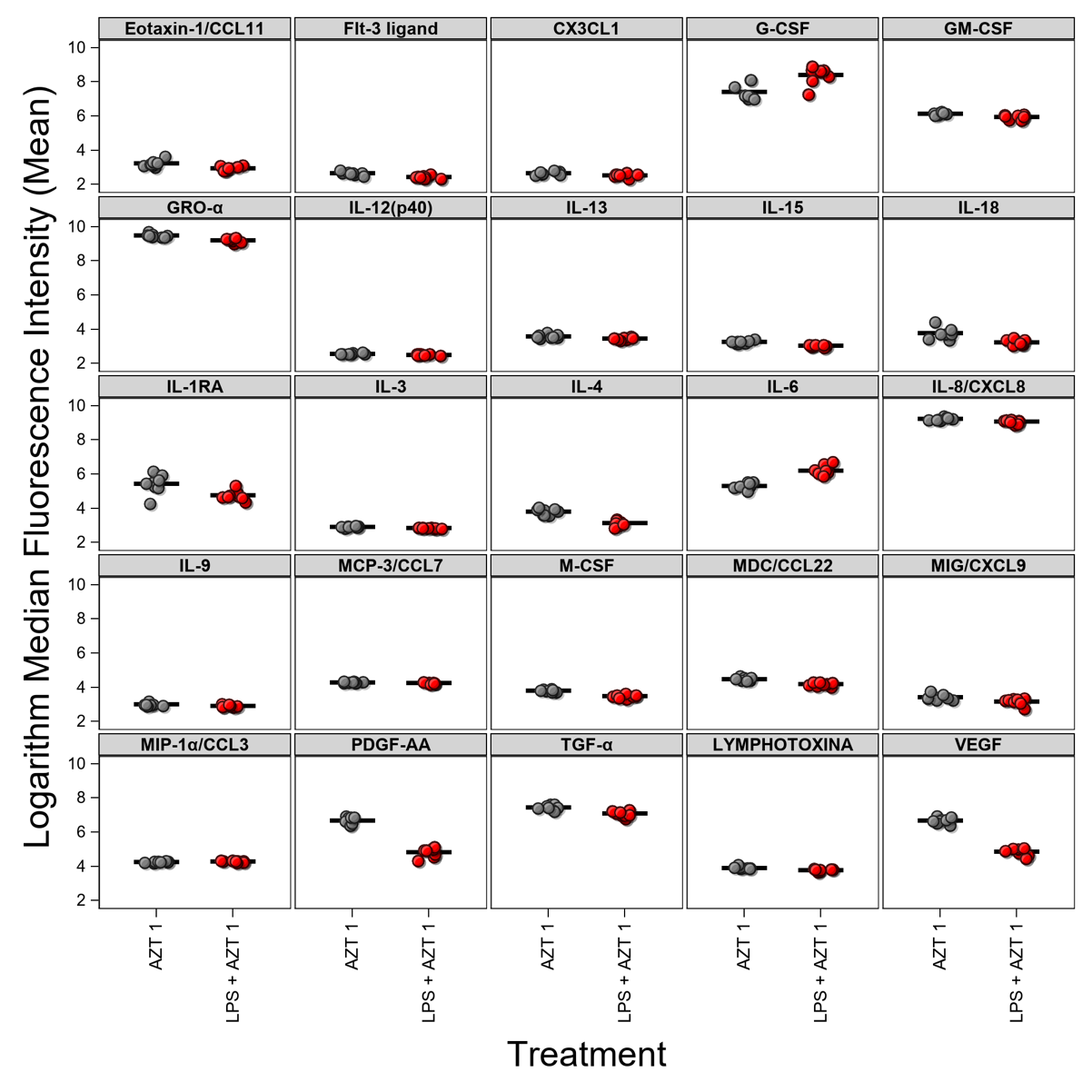


**Legends for the video supplementary material:**

**Video 1.** High-speed video microscopy recording ciliary beating of WT multi-ciliated cells.

**Video 2**. High-speed video microscopy recording of ciliary beating of DNAH5 multi-ciliated cells.

**Video 3**. High-speed video microscopy recording of ciliary beating of DNAH11 multi-ciliated cells.

**Video 4.** High-speed video microscopy recording of ciliary beating of CCDC39 multi-ciliated cells.

**Figure S4. Phase-contrast imaging of Incucyte® SX1 Live-Cell Analysis System for WT – Control.** Image of WT basal cell growth after 72 hours incubation.


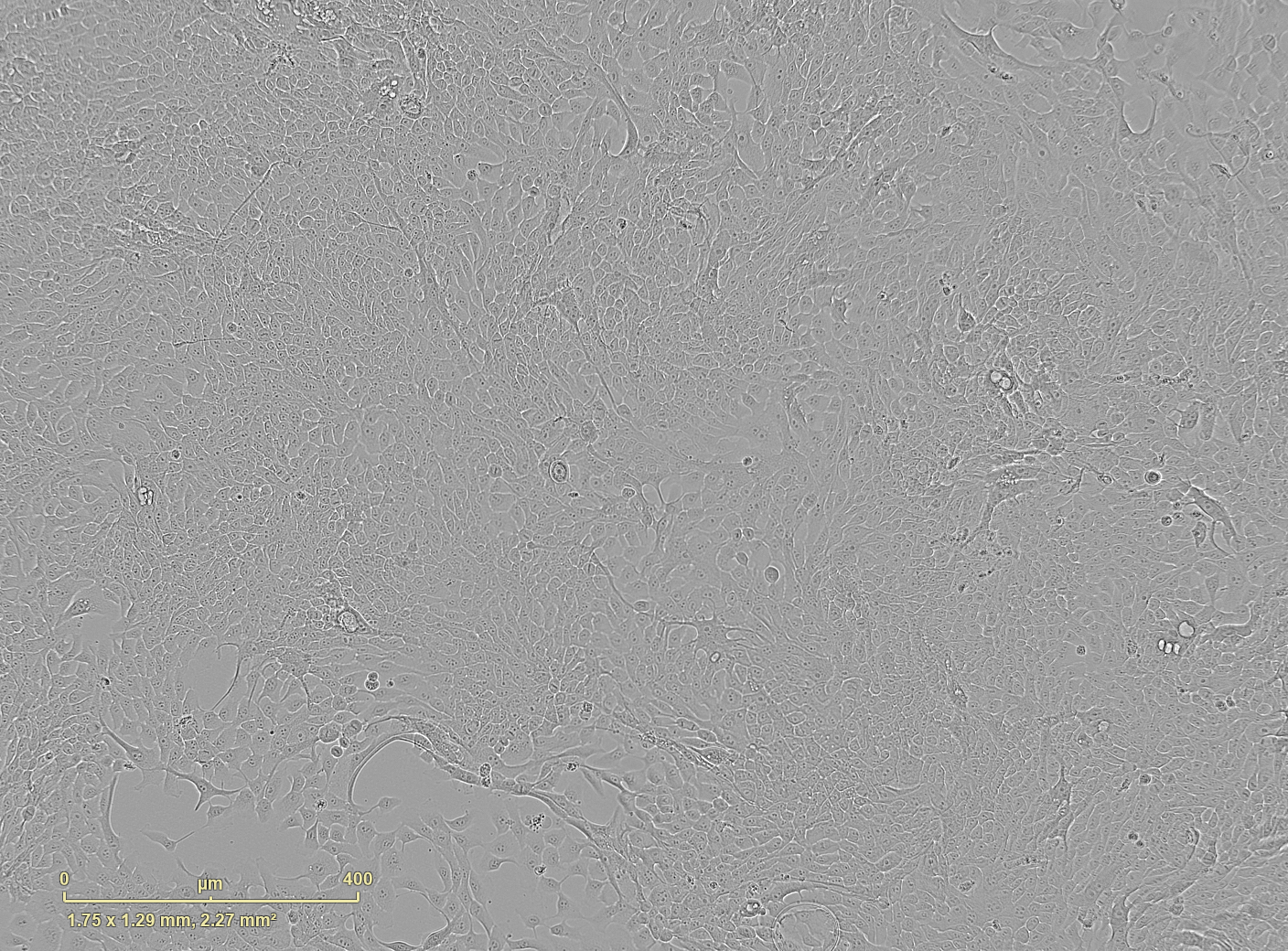


**Figure S5. Phase-contrast imaging of Incucyte® SX1 Live-Cell Analysis System for WT – AZT** **1 μg/l.** Image of WT basal cell growth after 72 hours incubation.


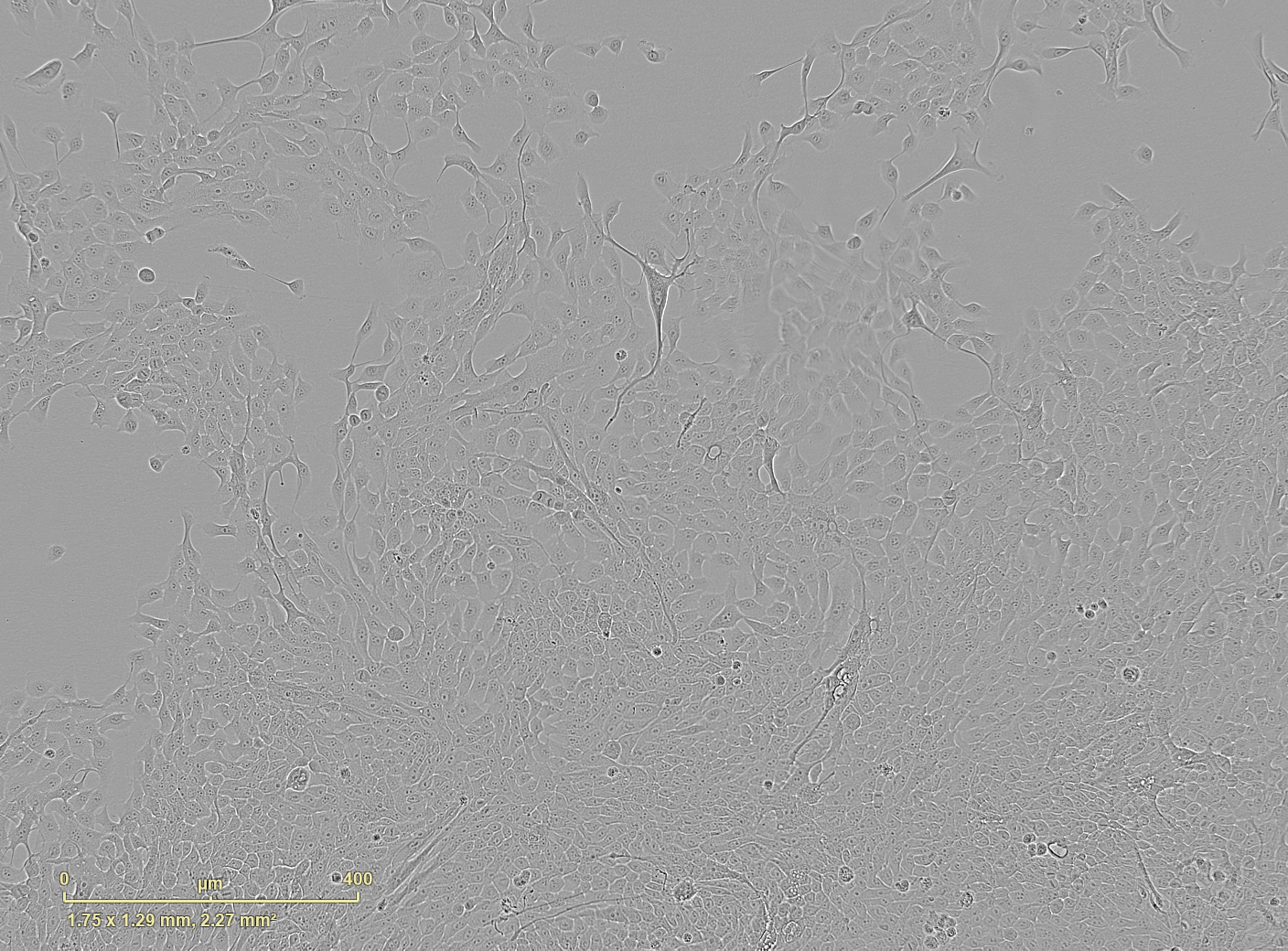


**Figure S6. Phase-contrast imaging of Incucyte® SX1 Live-Cell Analysis System for WT – AZT** **10 μg/l.** Image of WT basal cell growth after 72 hours incubation.


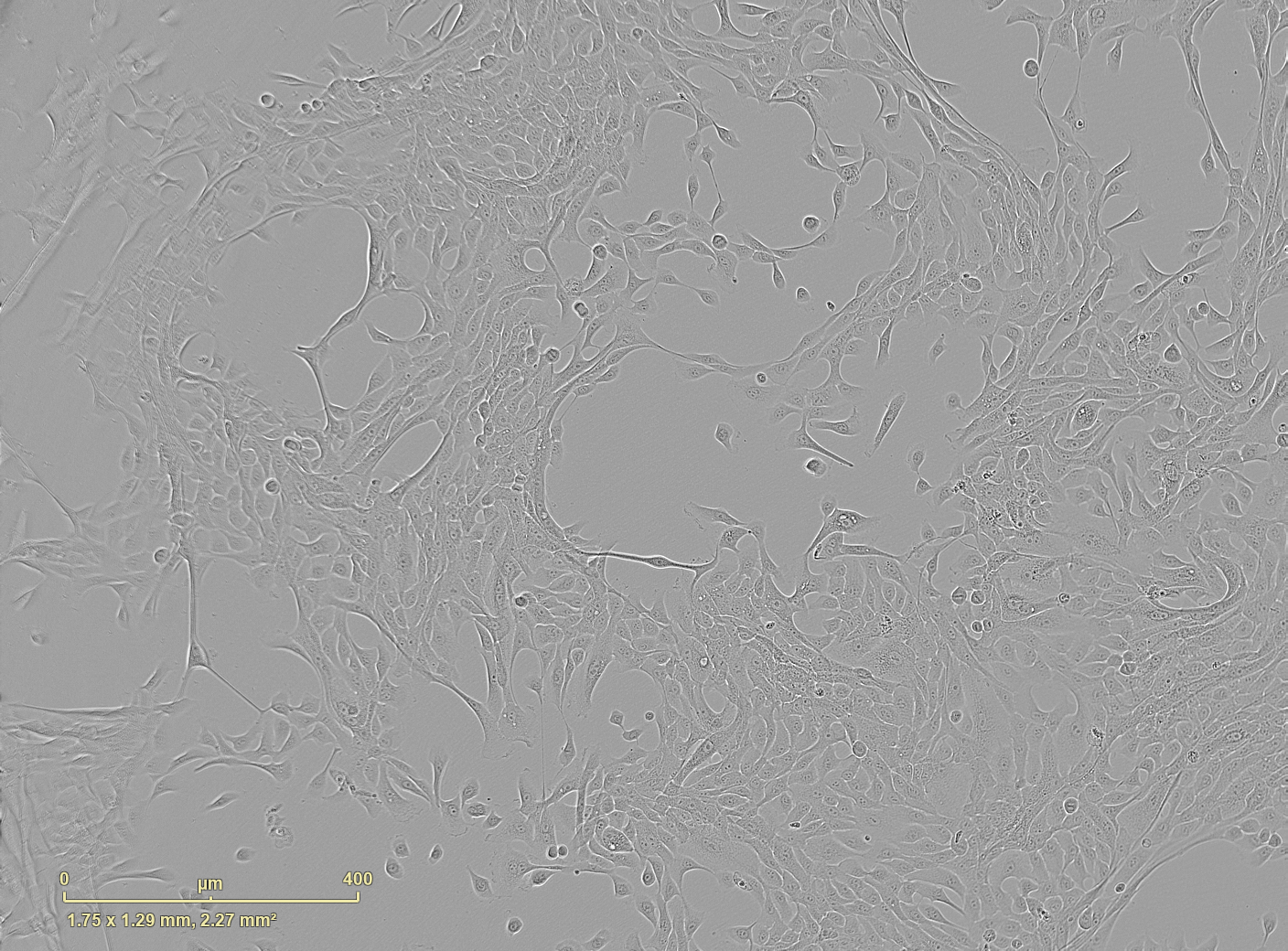


**Figure S7. Phase-contrast imaging of Incucyte® SX1 Live-Cell Analysis System for WT – LPS + AZT** **1 μg/l.** Image of WT basal cell growth after 72 hours incubation.


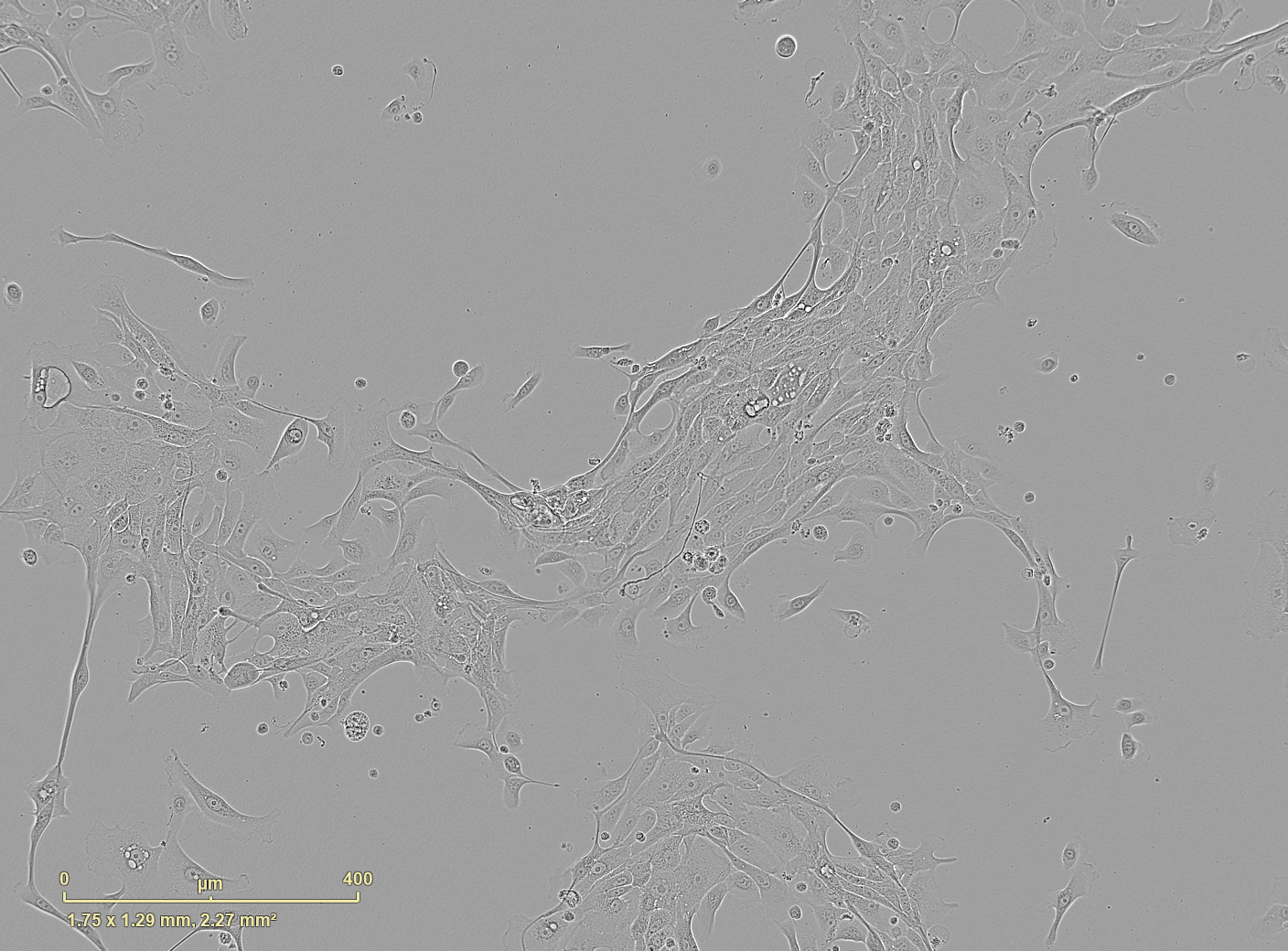


**Figure S8. Phase-contrast imaging of Incucyte® SX1 Live-Cell Analysis System for WT – LPS + AZT** **10 μg/l.** Image of WT basal cell growth after 72 hours incubation.


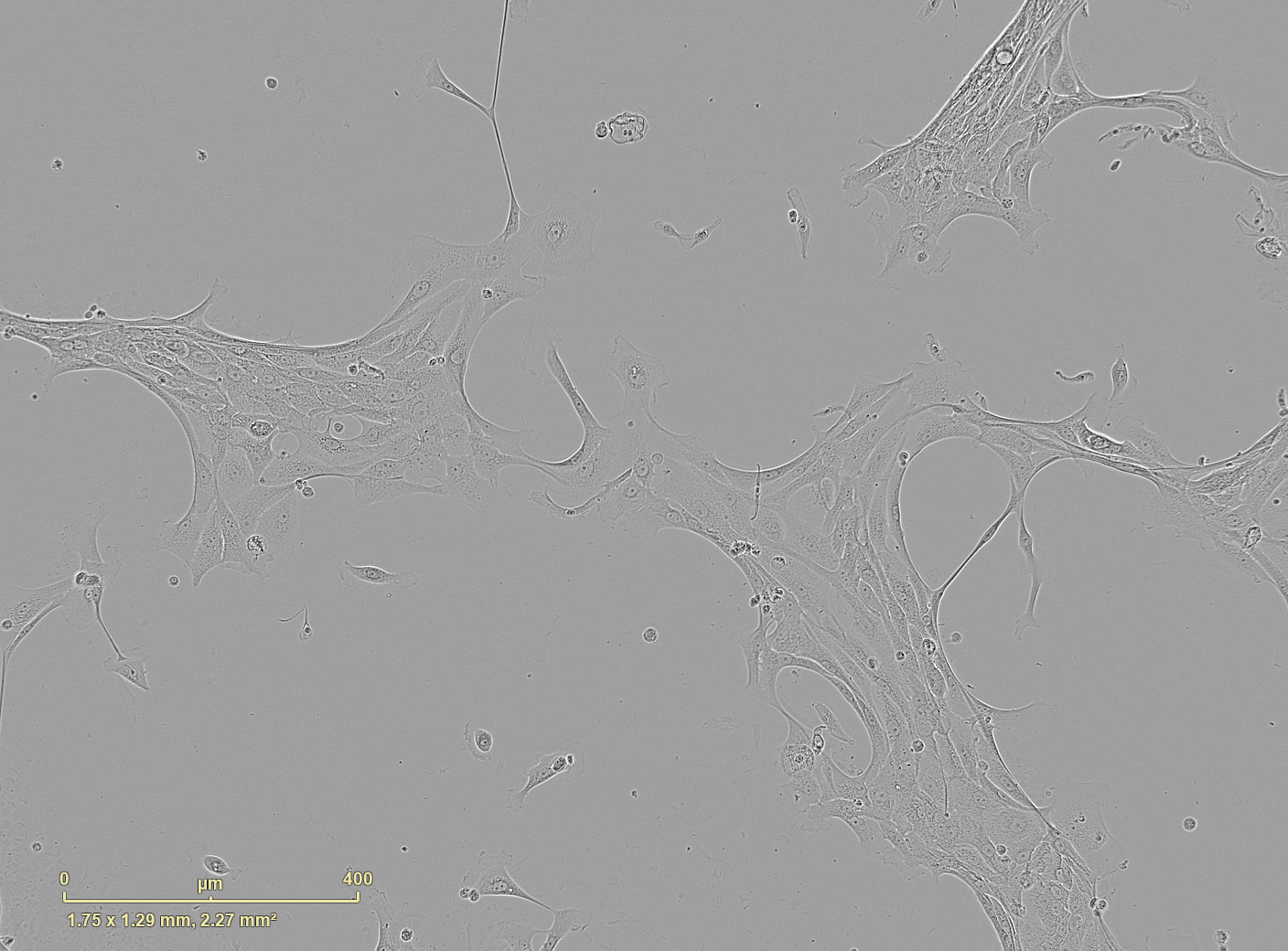


**Figure S9. Phase-contrast imaging of Incucyte® SX1 Live-Cell Analysis System for DNAH5 – Control.** Image of DNAH5 basal cell growth after 72 hours incubation.


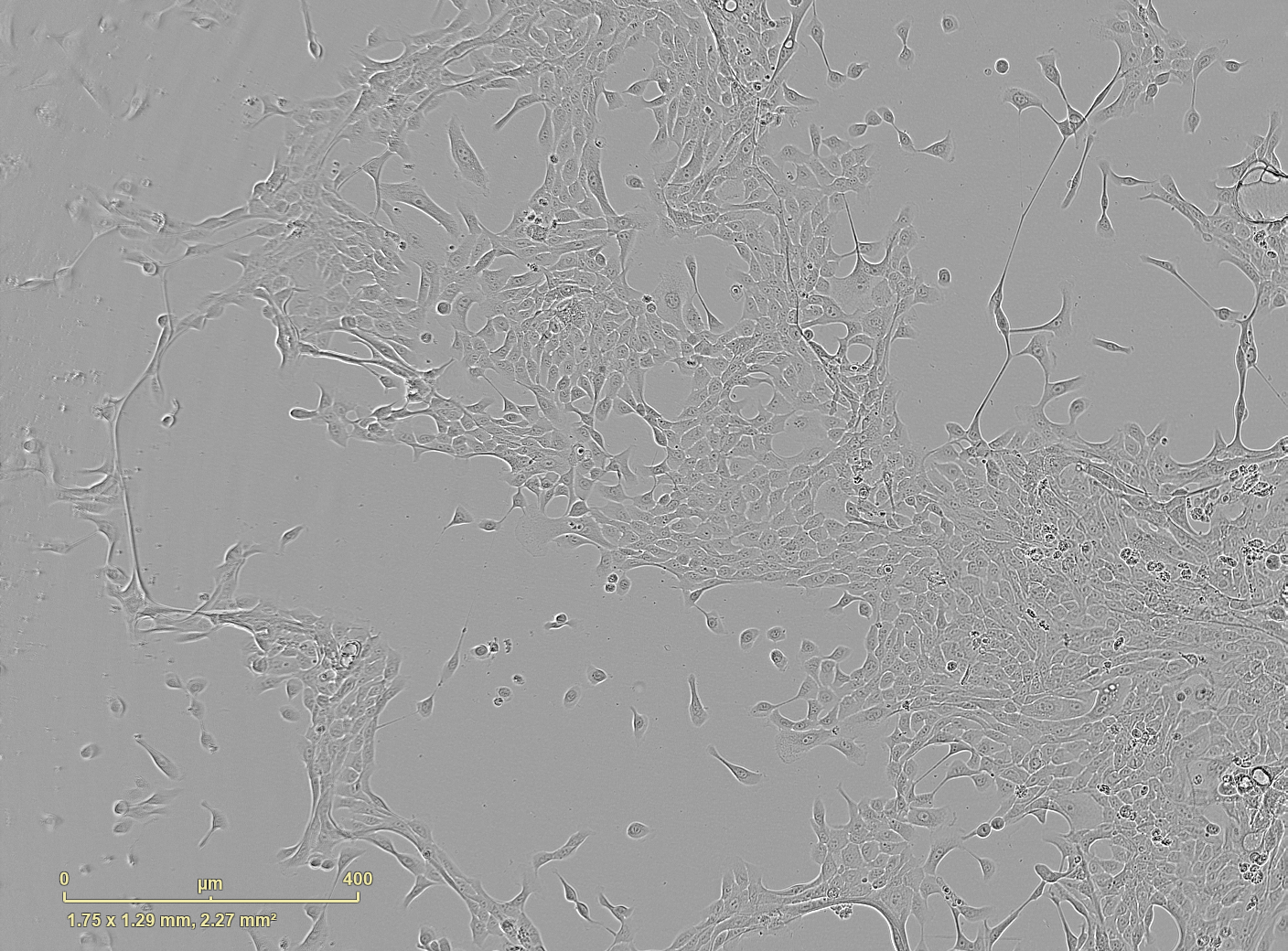


**Figure S10. Phase-contrast imaging of Incucyte® SX1 Live-Cell Analysis System for DNAH5 – AZT** **1 μg/l.** Image of DNAH5 basal cell growth after 72 hours incubation.


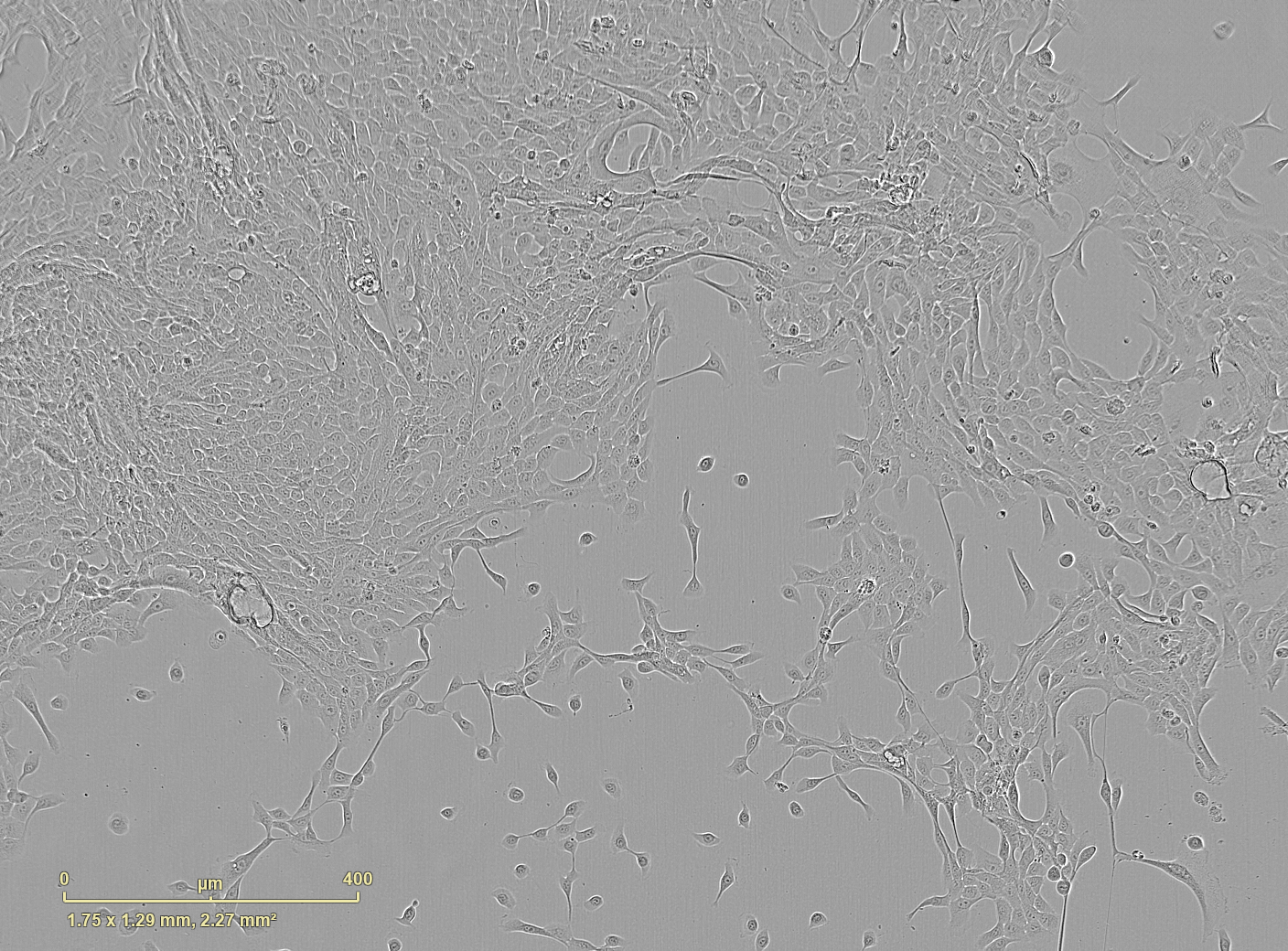


**Figure S11. Phase-contrast imaging of Incucyte® SX1 Live-Cell Analysis System for DNAH5 – AZT** **10 μg/l.** Image of DNAH5 basal cell growth after 72 hours incubation.


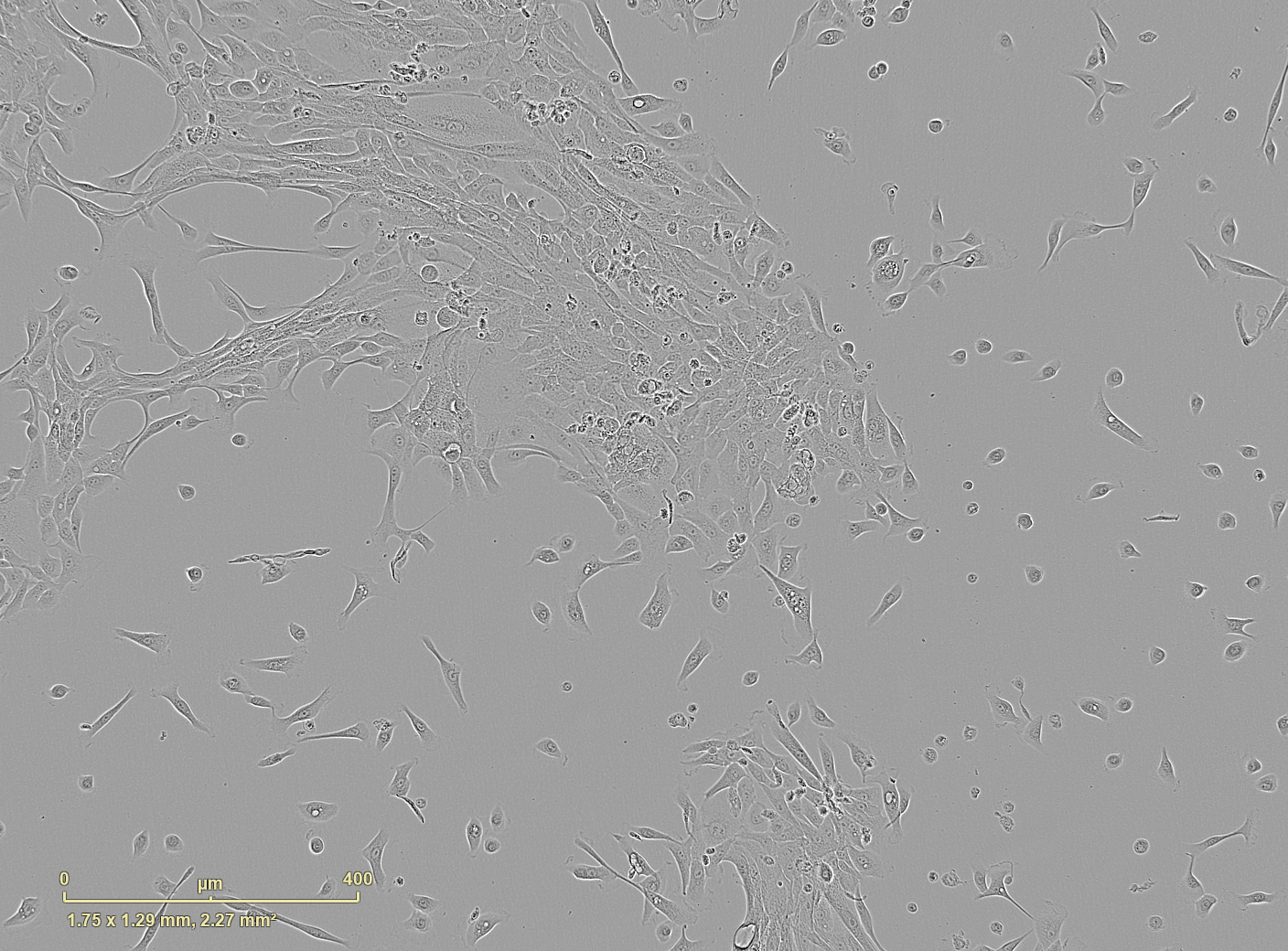


**Figure S12. Phase-contrast imaging of Incucyte® SX1 Live-Cell Analysis System for DNAH5 – LPS + AZT** **1 μg/l.** Image of DNAH5 basal cell growth after 72 hours incubation.


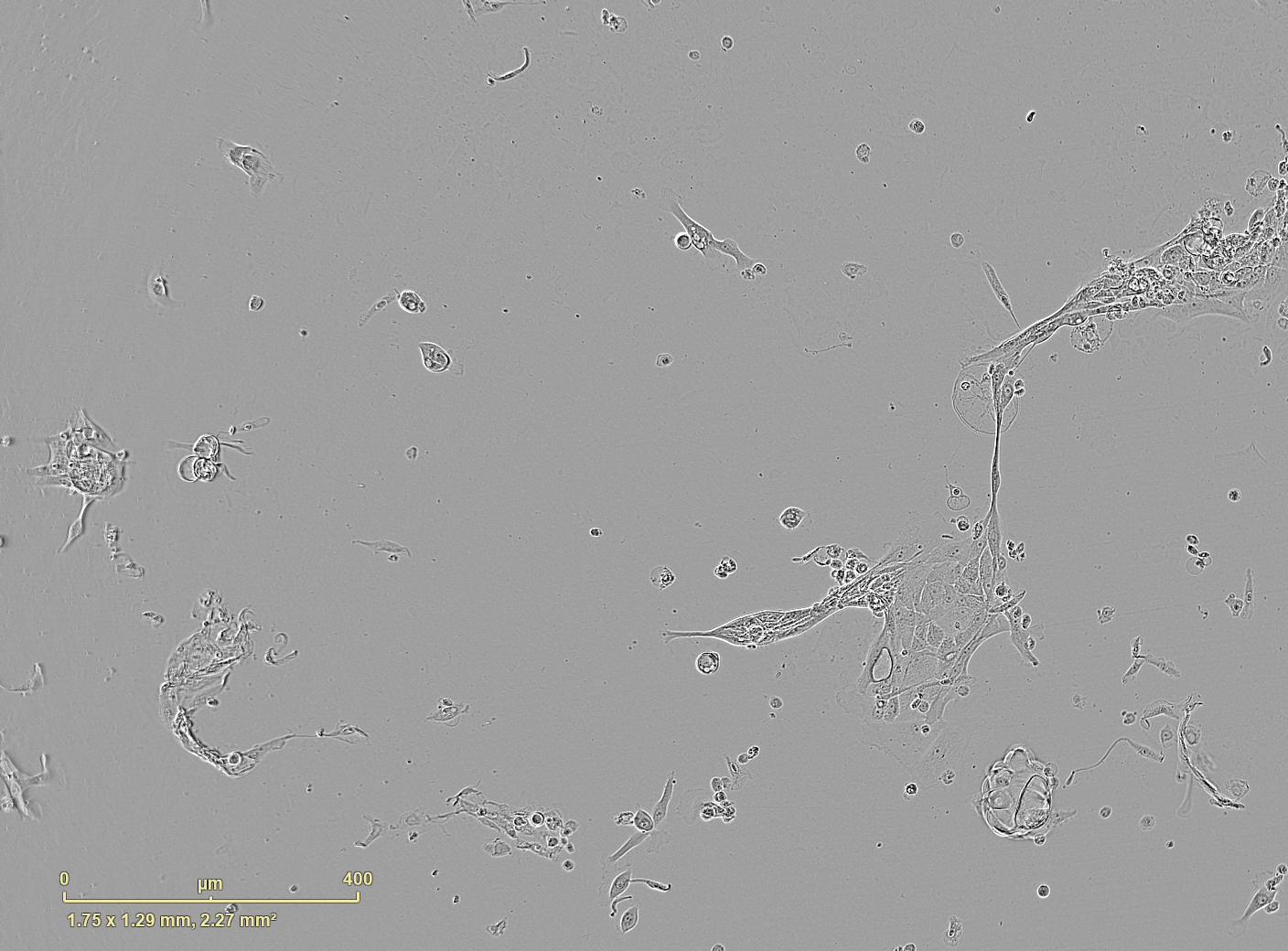


**Figure S13. Phase-contrast imaging of Incucyte® SX1 Live-Cell Analysis System for DNAH5 – LPS + AZT** **10 μg/l.** Image of DNAH5 basal cell growth after 72 hours incubation.


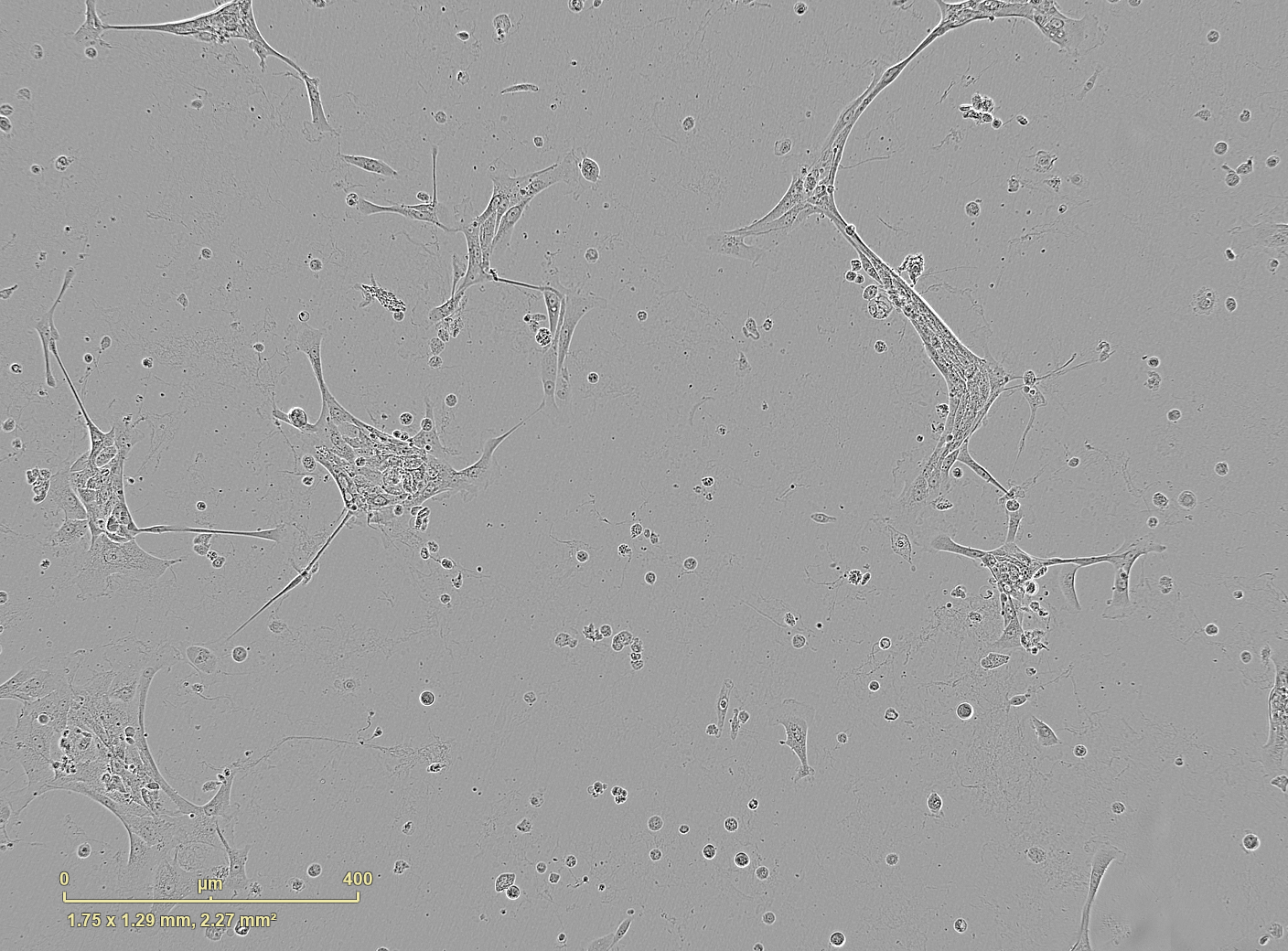


**Figure S14. Phase-contrast imaging of Incucyte® SX1 Live-Cell Analysis System for DNAH11 – Control.** Image of DNAH11 basal cell growth after 72 hours incubation.


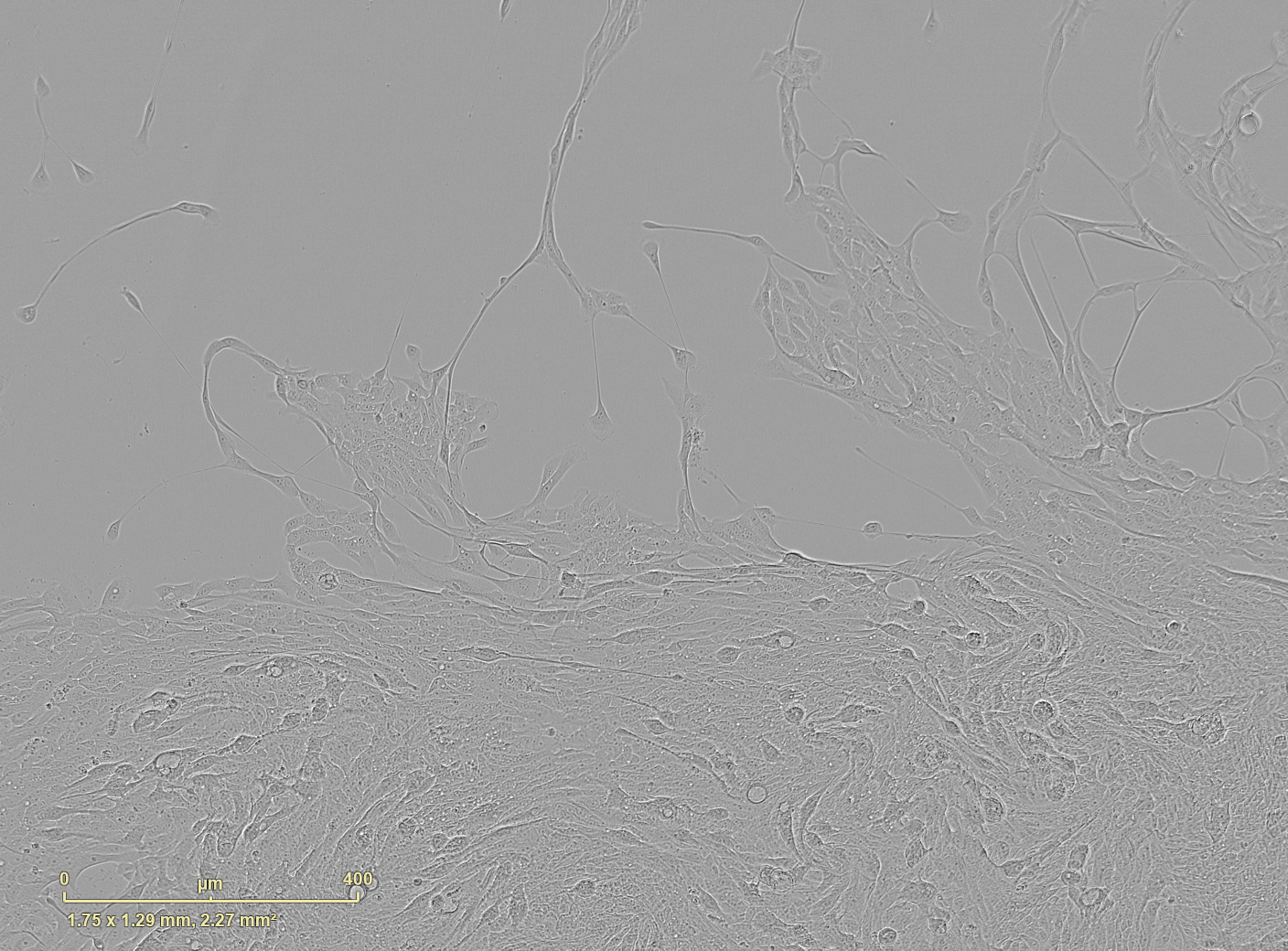


**Figure S15. Phase-contrast imaging of Incucyte® SX1 Live-Cell Analysis System for DNAH11 – AZT** **1 μg/l.** Image of DNAH11 basal cell growth after 72 hours incubation.


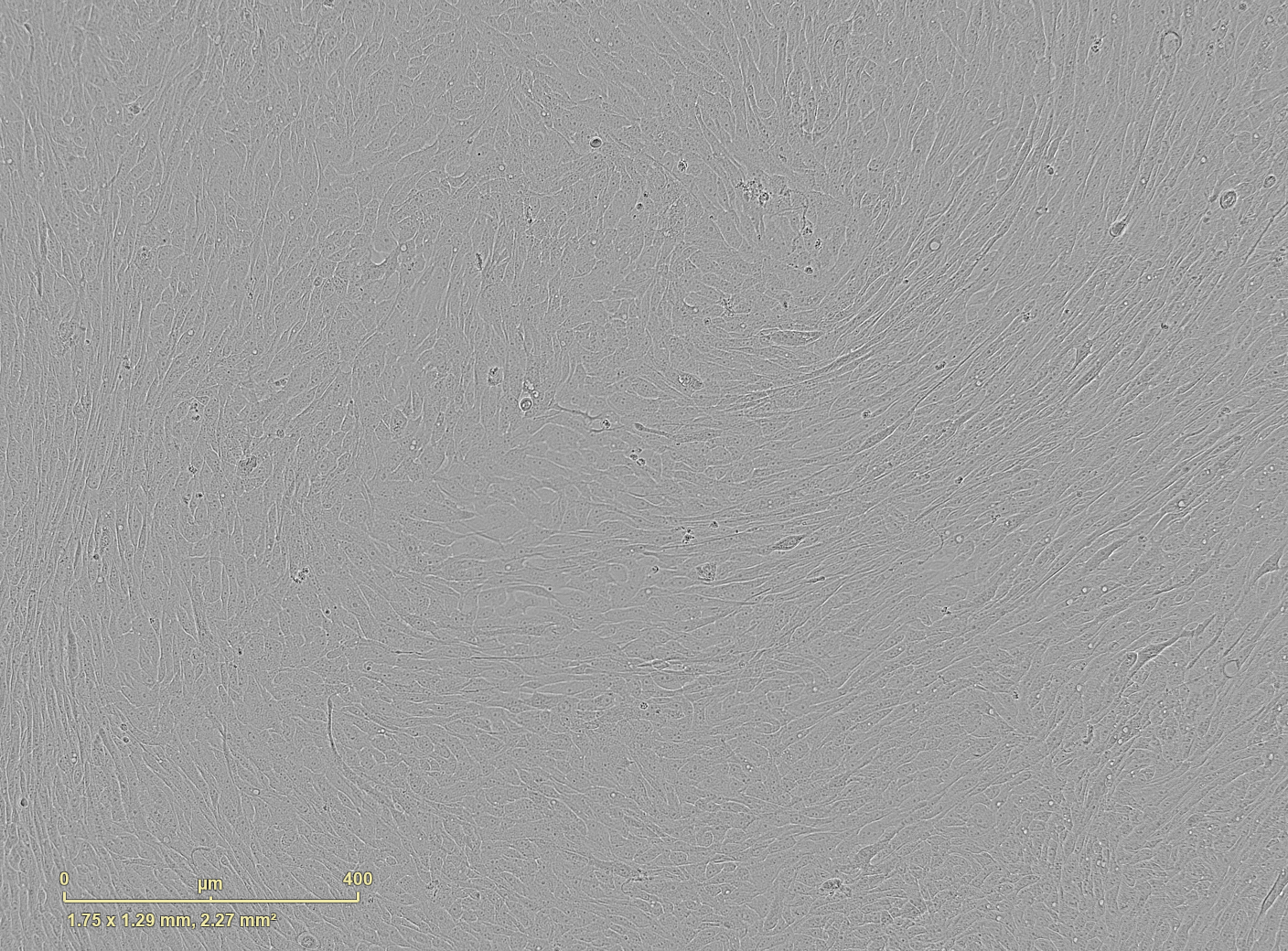


**Figure S16. Phase-contrast imaging of Incucyte® SX1 Live-Cell Analysis System for DNAH11 – AZT** **10 μg/l.** Image of DNAH11 basal cell growth after 72 hours incubation.


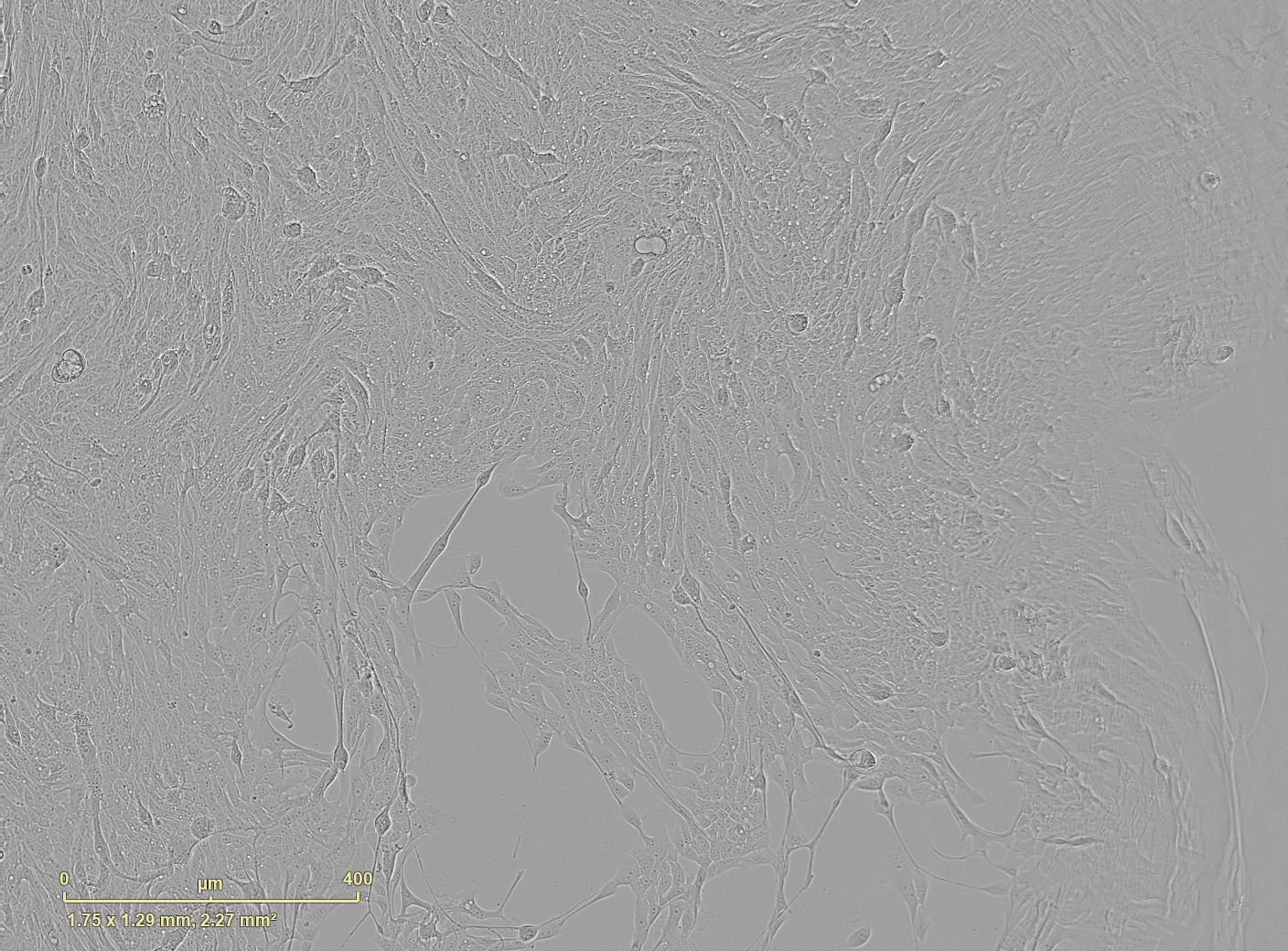


**Figure S17. Phase-contrast imaging of Incucyte® SX1 Live-Cell Analysis System for DNAH11 – LPS + AZT** **1 μg/l.** Image of DNAH11 basal cell growth after 72 hours incubation.


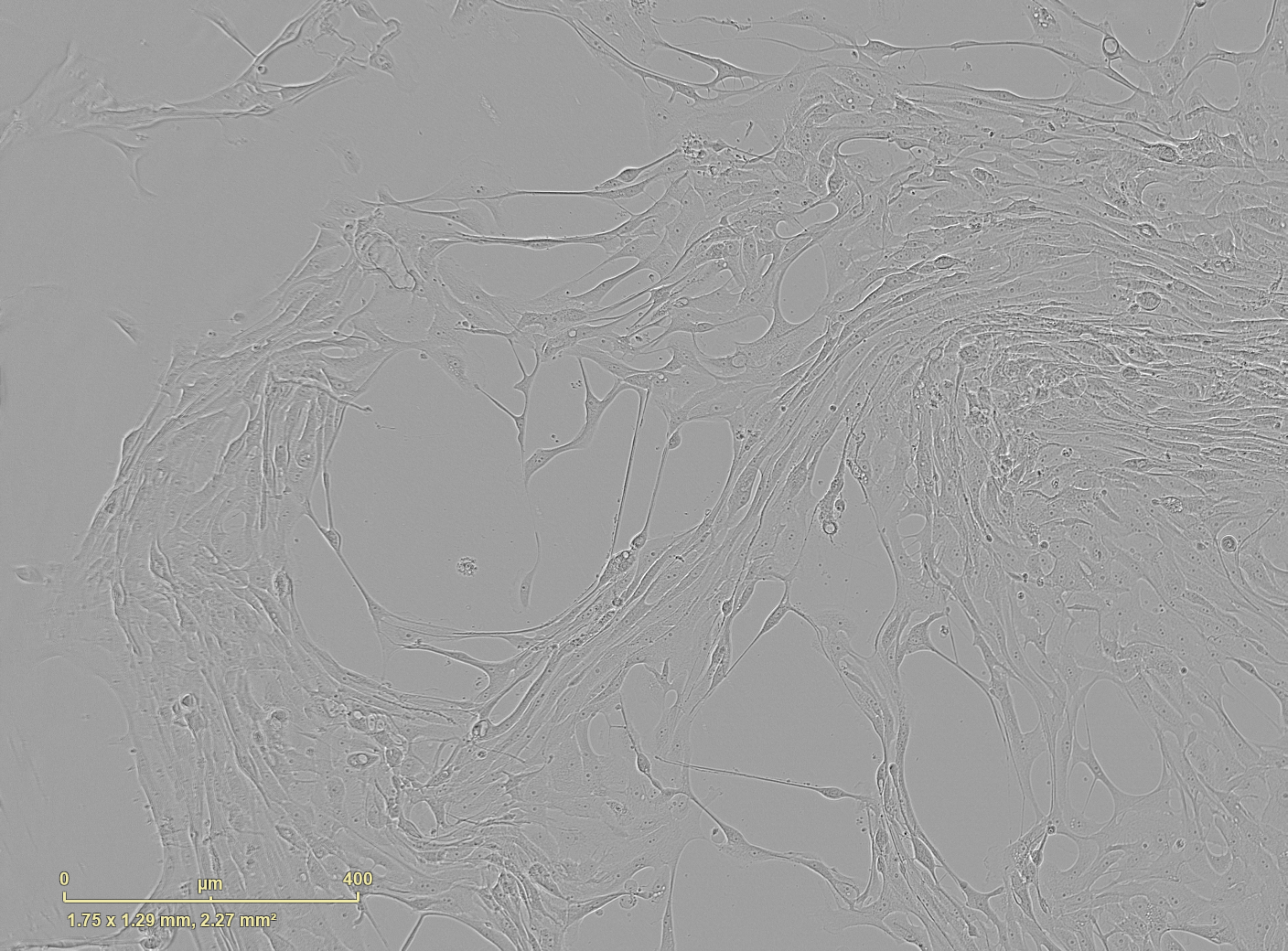


**Figure S18. Phase-contrast imaging of Incucyte® SX1 Live-Cell Analysis System for DNAH11 – LPS + AZT** **10 μg/l.** Image of DNAH11 basal cell growth after 72 hours incubation.


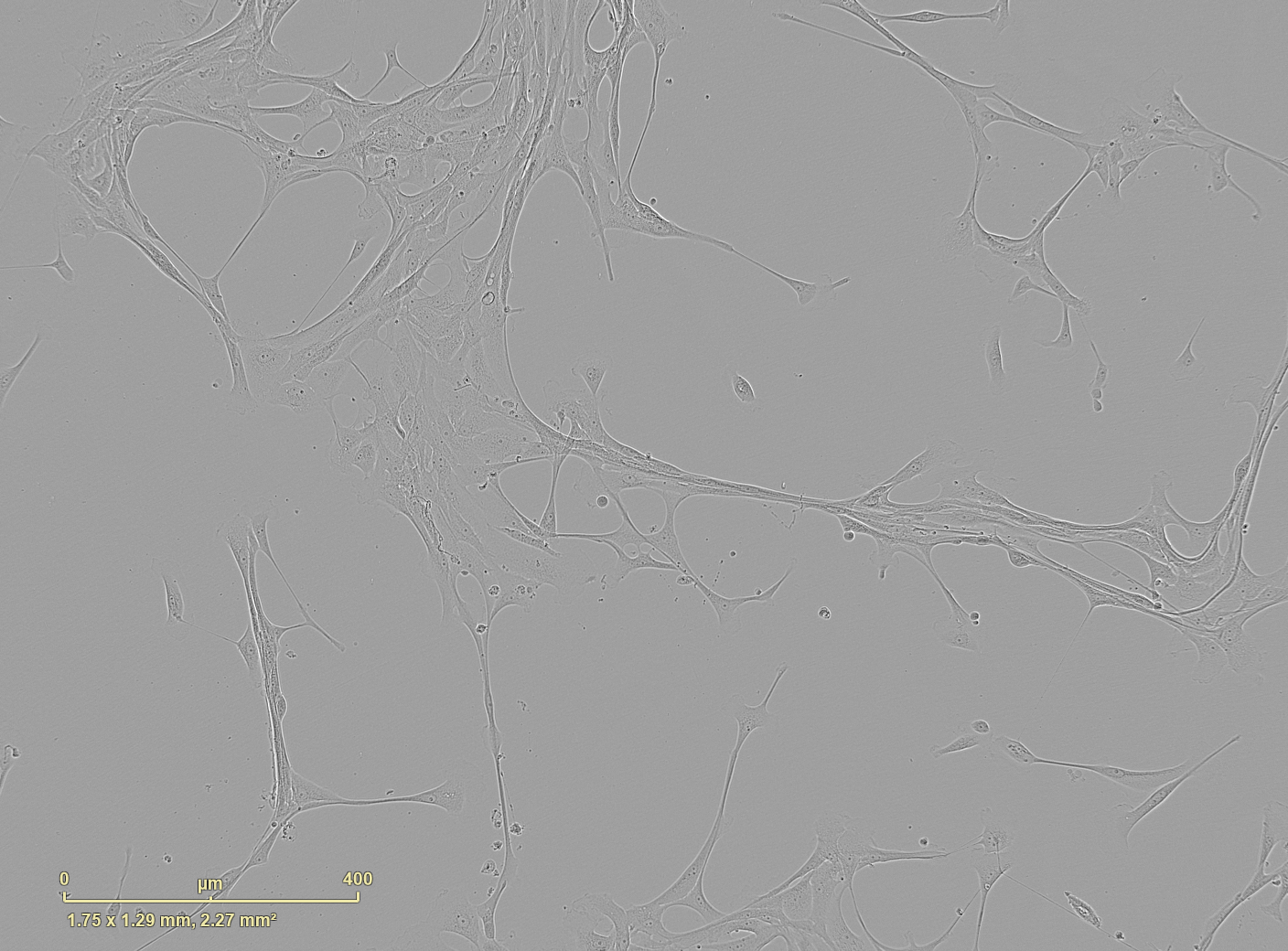


**Figure S19. Phase-contrast imaging of Incucyte® SX1 Live-Cell Analysis System for CCDC39 – Control.** Image of CCDC39 basal cell growth after 72 hours incubation.


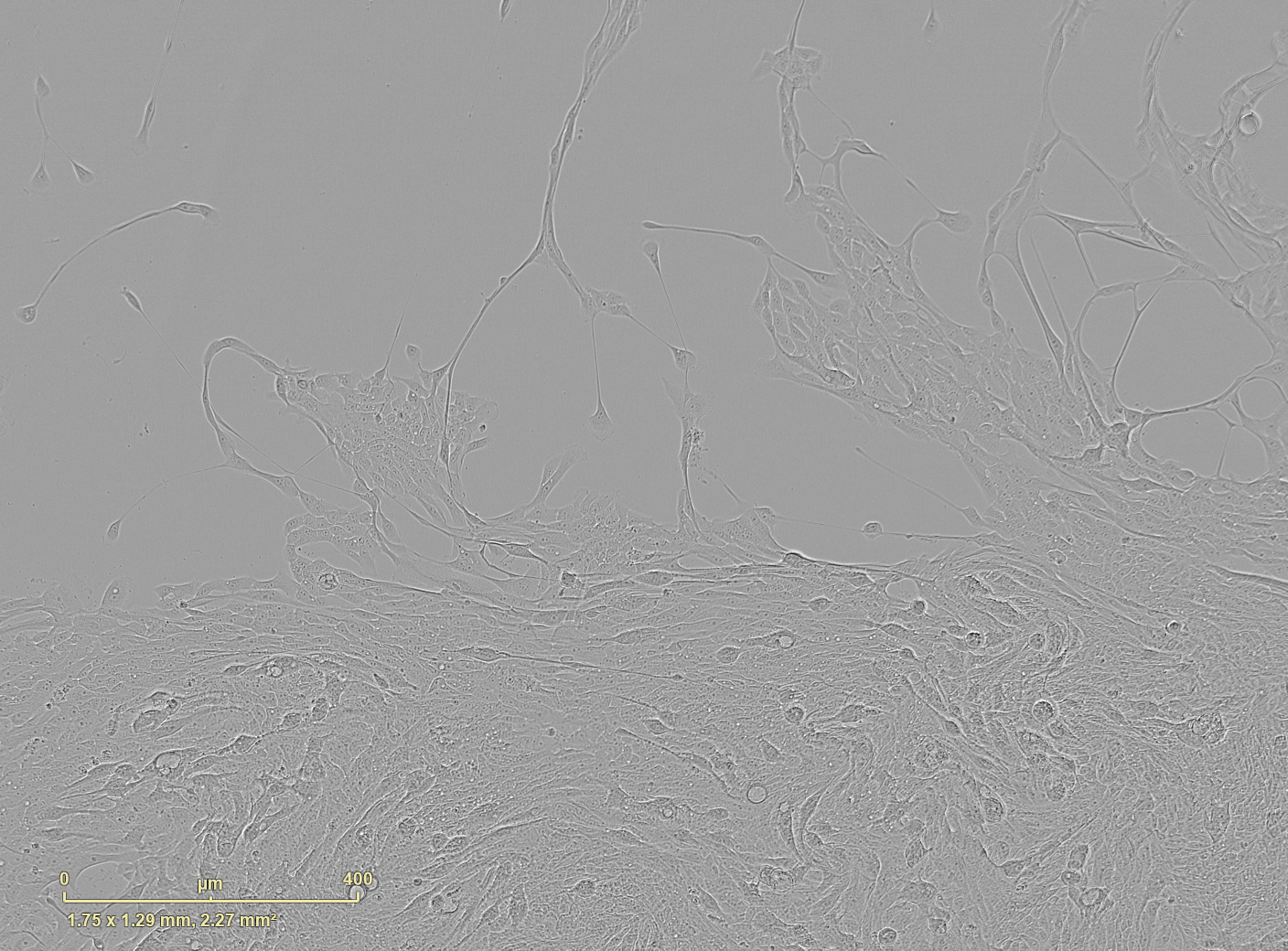


**Figure S20. Phase-contrast imaging of Incucyte® SX1 Live-Cell Analysis System for CCDC39 – AZT** **1 μg/l.** Image of CCDC39 basal cell growth after 72 hours incubation.


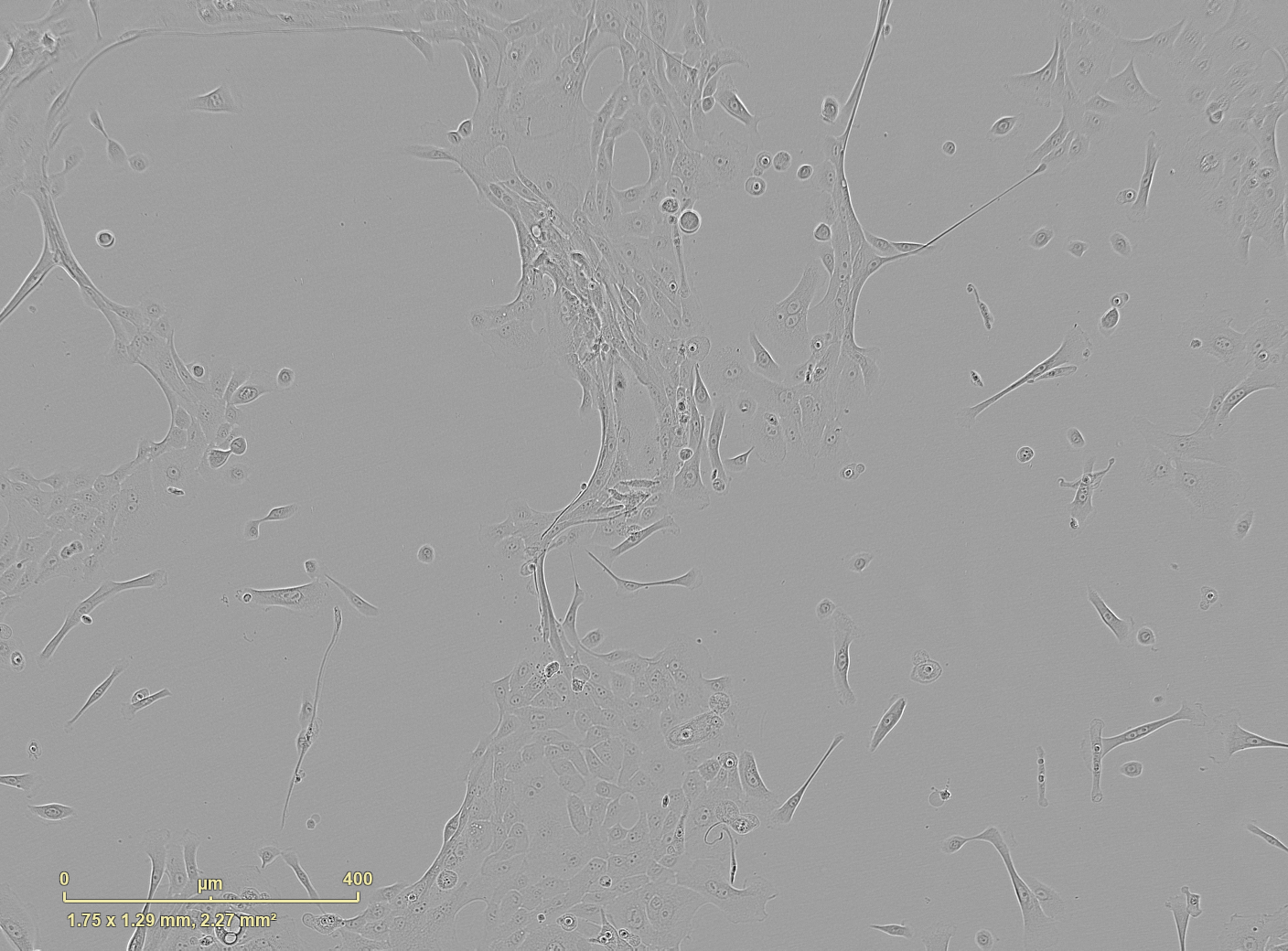


**Figure S21. Phase-contrast imaging of Incucyte® SX1 Live-Cell Analysis System for CCDC39 – AZT** **10 μg/l.** Image of CCDC39 basal cell growth after 72 hours incubation.


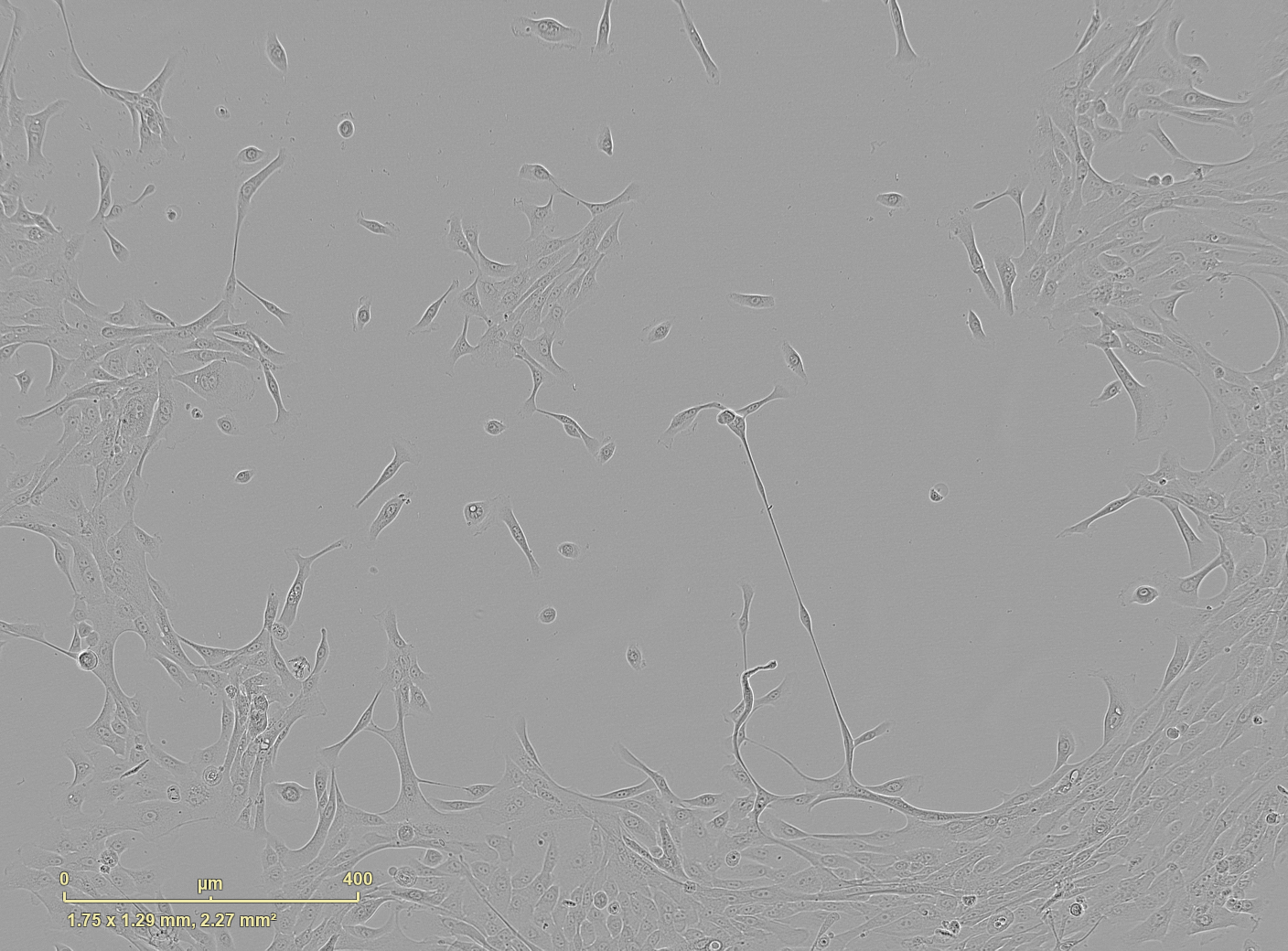


**Figure S22. Phase-contrast imaging of Incucyte® SX1 Live-Cell Analysis System for CCDC39 – LPS + AZT** **1 μg/l.** Image of CCDC39 basal cell growth after 72 hours incubation.


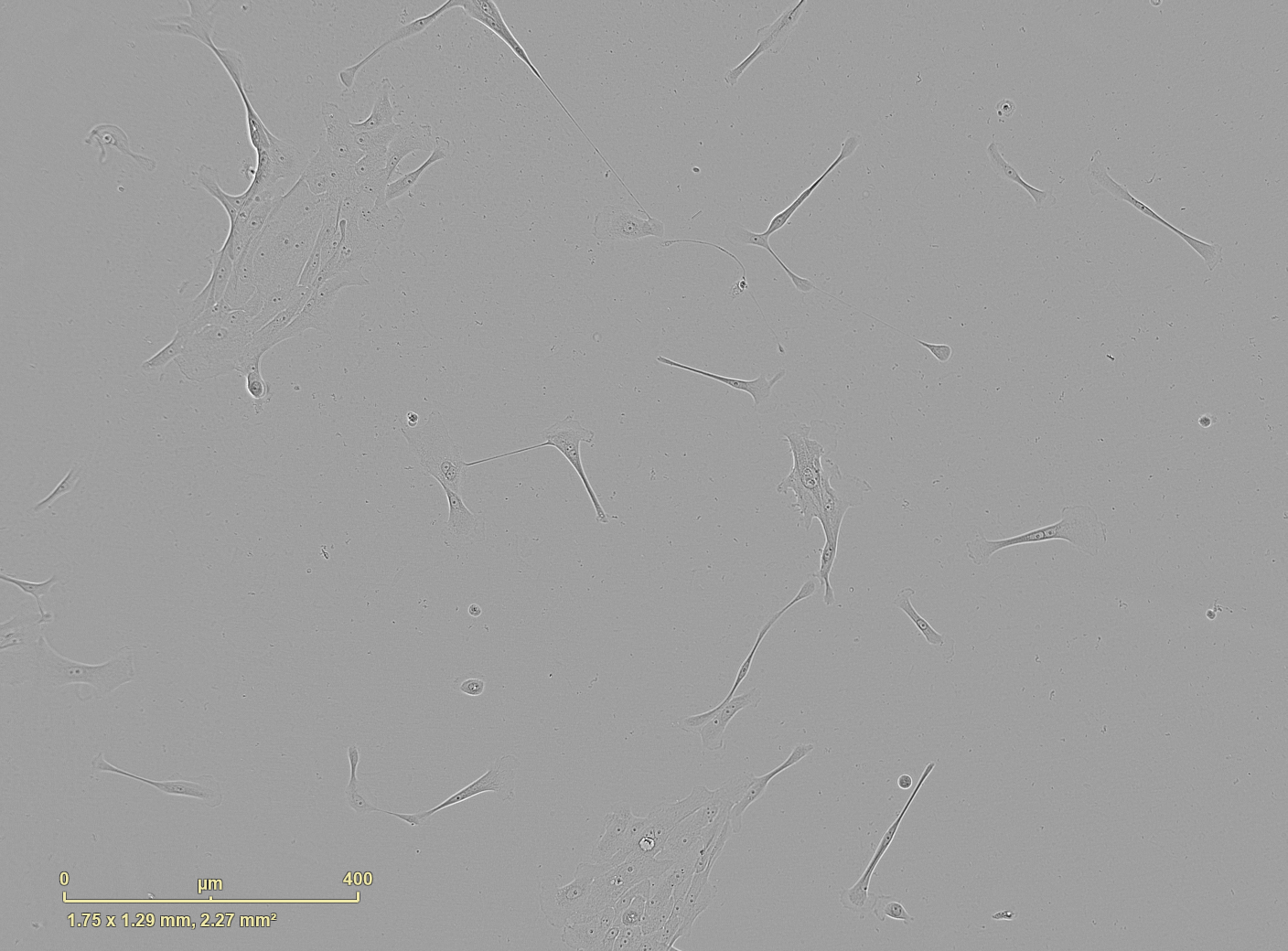


**Figure S23. Phase-contrast imaging of Incucyte® SX1 Live-Cell Analysis System for CCDC39 – LPS + AZT** **10 μg/l.** Image of CCDC39 basal cell growth after 72 hours incubation.


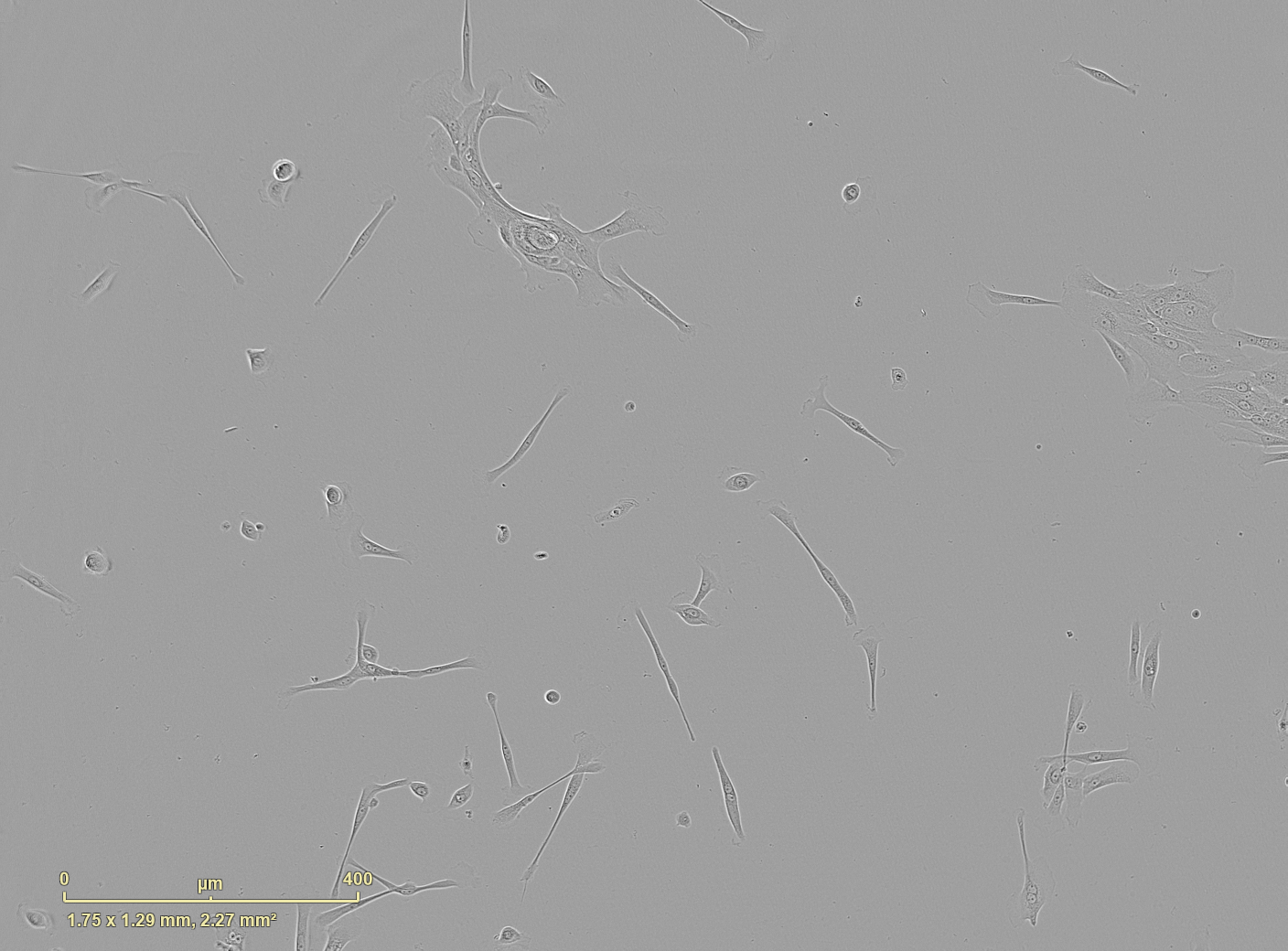

Supplement: Supplementary file 4 — Supplementary Information. [file 41598_2023_41577_MOESM4_ESM.docx]
